# Evolution toward beta common chain receptor usage links the matrix proteins of HIV-1 and its ancestors to human erythropoietin

**DOI:** 10.1073/pnas.2021366118

**Published:** 2020-12-28

**Authors:** Francesca Caccuri, Pasqualina D’Ursi, Matteo Uggeri, Antonella Bugatti, Pietro Mazzuca, Alberto Zani, Federica Filippini, Mario Salmona, Domenico Ribatti, Mark Slevin, Alessandro Orro, Wuyuan Lu, Pietro Liò, Robert C. Gallo, Arnaldo Caruso

**Affiliations:** ^a^Department of Molecular and Translational Medicine, University of Brescia Medical School, 25123 Brescia, Italy;; ^b^Institute of Technologies in Biomedicine, National Research Council, 20090 Segrate, Italy;; ^c^Department of Pharmacy, University of Genova, 16132 Genova, Italy;; ^d^Istituti di Ricovero e Cura a Carattere Assistenziale Istituto di Ricerche Farmacologiche Mario Negri, 20156 Milan, Italy;; ^e^Department of Basic Medical Sciences, Neurosciences and Sensory Organs, University of Bari Medical School, 70124 Bari, Italy;; ^f^School of Healthcare Science, Manchester Metropolitan University, M15GD Manchester, United Kingdom;; ^g^Institute of Human Virology, University of Maryland, Baltimore, MD 21201;; ^h^Department of Computer Science and Technology, University of Cambridge, CB3 0FD Cambridge, United Kingdom

**Keywords:** HIV-1 matrix protein p17, common beta chain receptor, human erythropoietin, HIV-1 and HIV-2 ancestors, HIV-1 evolutionary trajectory

## Abstract

The HIV-1 matrix protein p17 (p17) is a pleiotropic molecule impacting on different cell types. Its interaction with many cellular proteins underlines the importance of the viral protein as a major determinant of human specific adaptation. We previously showed the proangiogenic capability of p17. Here, by integrating functional analysis and receptor binding, we identify a functional epitope that displays molecular mimicry with human erythropoietin (EPO) and promotes angiogenesis through common beta chain receptor (βCR) activation. The functional EPO-like epitope was found to be present in the matrix protein of HIV-1 ancestors SIV originated in chimpanzees (SIVcpz) and gorillas (SIVgor) but not in that of HIV-2 and its ancestor SIVsmm from sooty mangabeys. According to biological data, evolution of the EPO-like epitope showed a clear differentiation between HIV-1/SIVcpz-gor and HIV-2/SIVsmm branches, thus highlighting this epitope on p17 as a divergent signature discriminating HIV-1 and HIV-2 ancestors. P17 is known to enhance HIV-1 replication. Similarly to other βCR ligands, p17 is capable of attracting and activating HIV-1 target cells and promoting a proinflammatory microenvironment. Thus, it is tempting to speculate that acquisition of an epitope on the matrix proteins of HIV-1 ancestors capable of triggering βCR may have represented a critical step to enhance viral aggressiveness and early human-to-human SIVcpz/gor dissemination. The hypothesis that the p17/βCR interaction and βCR abnormal stimulation may also play a role in sustaining chronic activation and inflammation, thus marking the difference between HIV-1 and HIV-2 in term of pathogenicity, needs further investigation.

## Significance

Immune activation and inflammation are predictors of serious non-AIDS events even in virally suppressed HIV-1−infected individuals. This does not apply to HIV-2−infected patients, who experience a form of attenuated HIV-1 disease. Here, we show that the HIV-1 matrix protein 17 (p17) binds to and activates the common beta chain receptor (βCR). The βCR-activating epitope on p17 is expressed on the matrix protein of HIV-1 ancestors but not on that of HIV-2 and its ancestors. Our finding highlights this epitope as a signature tracing the HIV-1 evolutionary trajectory that may have represented a critical step to enhance the HIV-1 ancestors aggressiveness and early human-to-human transmission. Whether this functional epitope actually marks the pathogenic difference between HIV-1 and HIV-2 needs further investigation.

HIV type 1 (HIV-1) has evolutionarily modified its proteins to better adapt to the human host and achieve a higher replication and transmission efficiency (1). The HIV-1 matrix protein p17 (p17) is particularly interesting being endowed with different biological activities within the virus particle. It plays a crucial role in both early and late stages of the virus life cycle (2). Within the mature virion, the majority of p17 molecules are located along the inner leaflet of the viral membrane (3). These molecules are believed to be important in assisting in early events such as uncoating and reverse transcription (4, 5). The biological activity of p17 was also found to occur at both the intracellular and extracellular level. Because of its polybasic stretches, p17 is suggested to take part to nuclear import of the HIV-1 preintegration complex (6). Following integration into the DNA of the host cell and expression of the viral genes, p17 exists as part of a larger precursor polyprotein encoded by the HIV-1 *Gag* gene (Pr55^*Gag*^). Via its myristoylated group, p17 targets Pr55^*Gag*^ molecules to the plasma membrane where it forms trimers essential for the packaging of the envelope glycoproteins (6). Interestingly, HIV-1–infected cells can unconventionally secrete p17 following Pr55^*Gag*^ binding to phosphatidylinositol 4, 5-bisphosphate and p17 cleavage from Pr55^*Gag*^ (7). Secretion of p17 occurs also in the absence of viral protease since its cleavage from Pr55^*Gag*^ can be operated, at the plasma membrane level, by cellular aspartyl proteases (7). Extracellularly, p17 has been found to deregulate the biological activity of many different immune cells that are directly or indirectly involved in AIDS pathogenesis. In particular, p17 triggers production and release of several inflammatory cytokines (8, 9) and enhances HIV-1 replication (9). At the same time, p17 is able to chemoattract B cells (10) and monocytes (11) and promote the release of proinflammatory chemokines directly linked to HIV-1 pathogenesis (11).

All p17 intracellular functions occurs following its interaction with as many as 20 different cellular proteins (12, 13). Extracellularly, p17 exerts its activity after binding to heparan sulfate proteoglycans (14), to the chemokine receptors CXCR1 and CXCR2, the physiological receptors for interleukin (IL)-8 (15, 16), to thrombin receptor (17) and possibly to other, still unknown, receptors (18, 19). Interaction of p17 with its receptors is followed by specific intracellular signaling pathways which are responsible for different biological activities (11, 15–19). It is therefore likely that certain sites on p17 are constrained to allow functional epitopes to perform unavoidable interactions aimed to favor a better HIV-1 replication and spreading.

Interaction of p17 with so many cellular proteins and receptors underlines the importance of the viral protein as a major determinant of human specific adaptation. Previous studies identified one site of p17 at the amino acid (aa.) position 30 (Gag30) that shifted from a Met or Leu expressed in simian immunodeficiency virus (SIV) derived from chimpanzees (*Pan troglodytes troglodytes*) (SIVcpz) and gorillas (*Gorilla gorilla gorilla*) (SIVgor) to Arg in inferred HIV-1 ancestors of group M, N, and O, and to Lys in many current pandemic strains (20, 21). Moreover, HIV-1 extensively passaged in chimpanzees were found to express Met at Gag30 and better replicate in chimpanzee CD4^+^ T cells than the original HIV-1 strains, while the opposite was shown in human CD4^+^ T cells (20) and human lymphoid tissue (21). All these results highlight that the p17 aa. position 30 is subjected to a strong host-specific selection pressure.

We previously showed the capability of p17 to promote angiogenesis and lymphangiogenesis in vitro and in vivo (16, 22). Surprisingly, by integrating functional analysis and receptor binding, here we identify two different functional epitopes which exert proangiogenic effects on human endothelial cells (ECs) by using different mechanisms and receptors. In particular, one of the two functional epitopes displays molecular mimicry with human erythropoietin (EPO) and exerts its biological activity by directly engaging the common beta chain receptor (βCR). Phylogenetic analysis underlined the presence of a functional EPO-like sequence in the matrix protein of HIV-1 ancestors SIVcpz and SIVgor, whereas it was not functioning in the matrix protein of HIV-2 and its ancestor SIVsmm from sooty mangabeys. The evolution of the EPO-like fragment shows a clear differentiation between HIV-1/SIVcpz-gor and HIV-2/SIVsmm branches, thus highlighting this epitope on p17 as a clear divergence signature discriminating HIV-1 and HIV-2 ancestors.

## Results

### P17 Possesses Two Functional Epitopes Promoting Angiogenesis and Lymphangiogenesis.

**Fig. 1.**
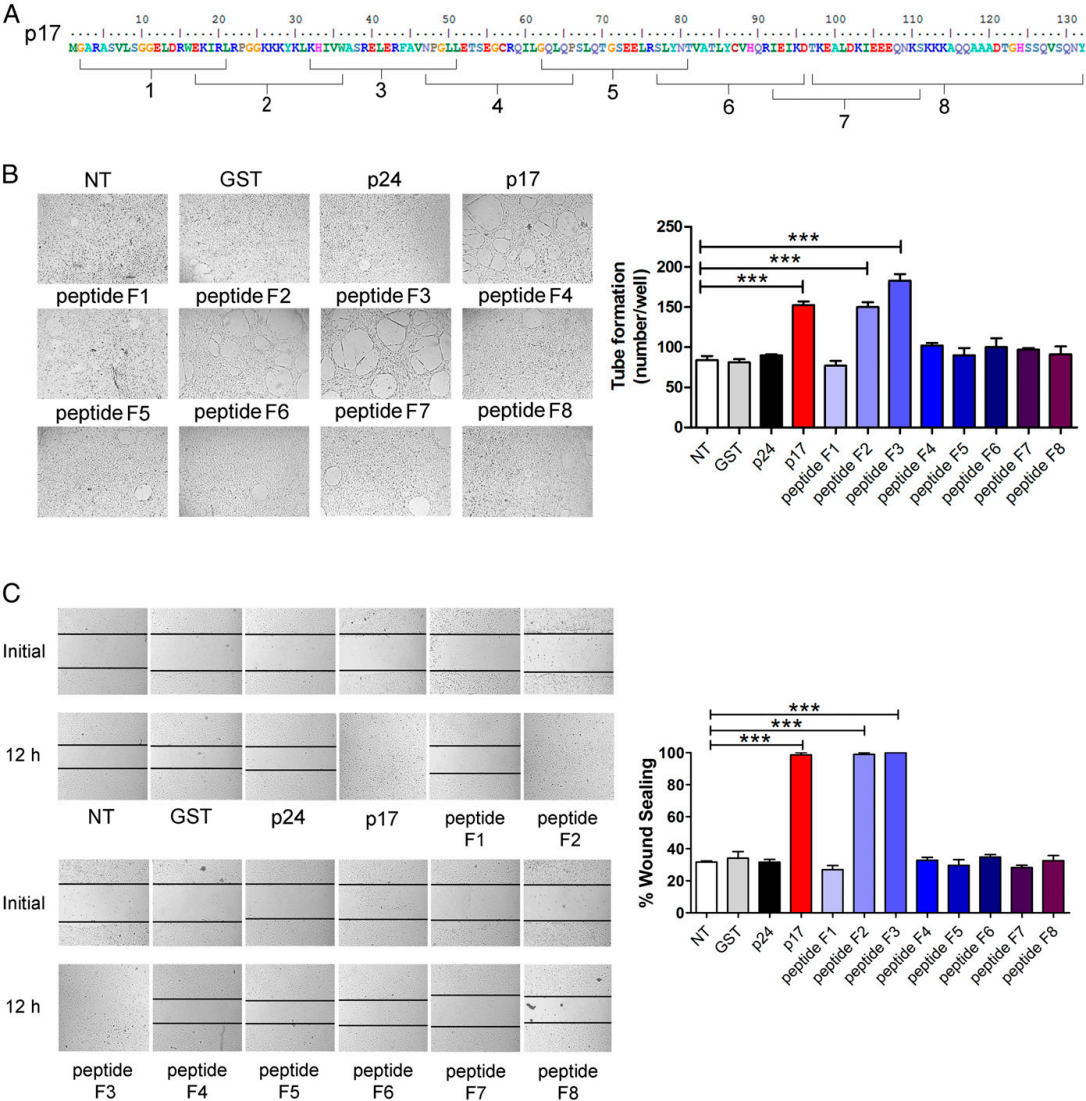
Ability of different p17-derived peptides to induce angiogenesis and migration on HUVECs. (*A*) aa. sequence of eight p17-derived peptides. (*B*) HUVECs were cultured under stressed condition (EBM containing 0.5% fetal bovine serum [FBS]) for 16 h at 37 °C and then stimulated for 8 h at 37 °C with 10 ng/mL GST, p24, p17, or each p17-derived peptide (F1, F2, F3, F4, F5, F6, F7, F8). NT, not treated. (*C*) HUVECs were cultured under stressed condition for 16 h at 37 °C, and then the confluent cell monolayers were scratched and stimulated for 12 h at 37 °C with medium alone (NT) or with medium containing 10 ng/mL GST, p24, p17, or each p17-derived peptide (F1, F2, F3, F4, F5, F6, F7, F8). Images are representative of three independent experiments with similar results. (Original magnification, 10×.) Data are the mean ± SD of one representative experiment, of three with similar results, performed in triplicate. Statistical analysis was performed by one-way ANOVA, and the Bonferroni post hoc test was used to compare data (***P < 0.001).

P17 displays a potent angiogenic and lymphangiogenic activity (16, 22, 23). To identify its functional epitope(s), we tested the ability of eight chemically synthesized p17-derived peptides (19) (Fig. 1*A*) to promote tube-like structure formation in vitro at the concentration of 10 ng/mL. HUVECs were nutrient starved for 16 h before seeding on 48-well plates (5 × 10^4^ per well) containing polymerized plugs of growth factor-reduced basement membrane extract. Activity screening identified two peptides of the eight, namely peptide F2 and F3, endowed with angiogenic activity (Fig. 1*B*). Similar results were obtained using human ECs derived from aorta and lung, and lymphatic ECs (LECs) derived from lymph node (*SI Appendix*, Fig. S1). Since p17 is known to promote EC migration (23), we tested the eight peptides for their capability to promote wound sealing. As shown in Fig. 1*C*, not-treated (NT) HUVECs reached a level of 29.5 ± 5.5% healing after 12 h of culture following scratch injury of the cell monolayer, whereas 100% sealing was observed in HUVECs treated with 10 ng of peptide F2 and F3. All the other tested peptides did not show any capability in promoting EC migration.

Recently, we demonstrated that peptide F2 was able to bind to CXCR1 and CXCR2 (19). As expected, the neutralizing monoclonal antibodies (mAb) to CXCR1 and CXCR2 were inhibitory toward peptide F2-induced angiogenesis. However, they did not impair the angiogenic activity of peptide F3 (*SI Appendix*, Fig. S2). These results suggest that peptide F3 uses a different receptor(s) than CXCR1 and CXCR2 to exert its angiogenic activity. The vasculogenic activity of peptide F2 and F3 was then investigated using the aortic ring assay (16). As shown in *SI Appendix*, Fig. S3*A*, the number of microvessels was significantly lower in vehicle alone phospate buffered saline (PBS) (11 ± 1) than in rings treated with 10 ng/mL peptide F2 (55 ± 5) or F3 (46 ± 8). The vasculogenic property of peptide F2 and F3 was additionally scrutinized in vivo by using the chick chorioallantoic membrane (CAM) assay (16). As shown in *SI Appendix*, Fig. S3*B*, a significant angiogenic response in the form of numerous allantoic neovessels developing radially toward the implant in a “spoke-wheel” was promoted by 50 ng/mL peptide F2 and F3 (mean number of vessels: 27 ± 4 and 30 ± 3, respectively) as compared to the vehicle alone (PBS) (mean number of vessels of 12 ± 2).

### Peptide F3 Displays Angiogenic Activity under Stressed and Normal Culture Conditions.

We previously demonstrated that p17 induces angiogenic and lymphangiogenic activity in its monomeric form and upon stress condition only (16, 22, 23). In order to understand if serum starvation is a condition sine qua non for sustaining the peptides’ angiogenic activity, we performed a tube-like structure formation experiment using HUVECs cultured under normal or stressed—serum deprived—conditions. Under stressed condition, HUVECs were highly susceptible to stimulation with p17 and with both peptide F2 and F3 (*SI Appendix*, Fig. S4*A*). Surprisingly, when the angiogenic assay was performed on HUVECs cultured under normal condition, peptide F3 was the only one able to promote angiogenesis (*SI Appendix*, Fig. S4*B*). Altogether, our data demonstrate that p17 has two different angiogenic epitopes: one acting by binding to CXCR1 and CXCR2 and promoting angiogenesis under stress condition only; the other interacting with a still-unknown receptor promoting angiogenesis under both stressed and normal cell culture conditions. In a previous study, we showed that peptide F3 is functional on oligomerized p17 (24). According to this evidence, we tested the angiogenic activity of monomeric and oligomeric p17 on HUVECs cultured under normal condition. As expected, monomeric p17 did not promote angiogenesis, whereas the oligomeric p17 did promote it (*SI Appendix*, Fig. S4*C*). At the same time, either monomeric or oligomeric p17s were able to promote angiogenesis on HUVECs under stressed culture condition (*SI Appendix*, Fig. S4*D*). The angiogenic activity of monomeric p17 is autophagy-dependent (23). As shown in *SI Appendix*, Fig. S4 *E* and *F*, the angiogenic activity of the peptide F2, but not F3, was found to be inhibited by 3-methyladenine (3-MA), a pharmacological inhibitor of autophagy, and by silencing of Beclin-1, a protein required for autophagosome formation.

### Mimicry between p17 and EPO.

**Fig. 2.**
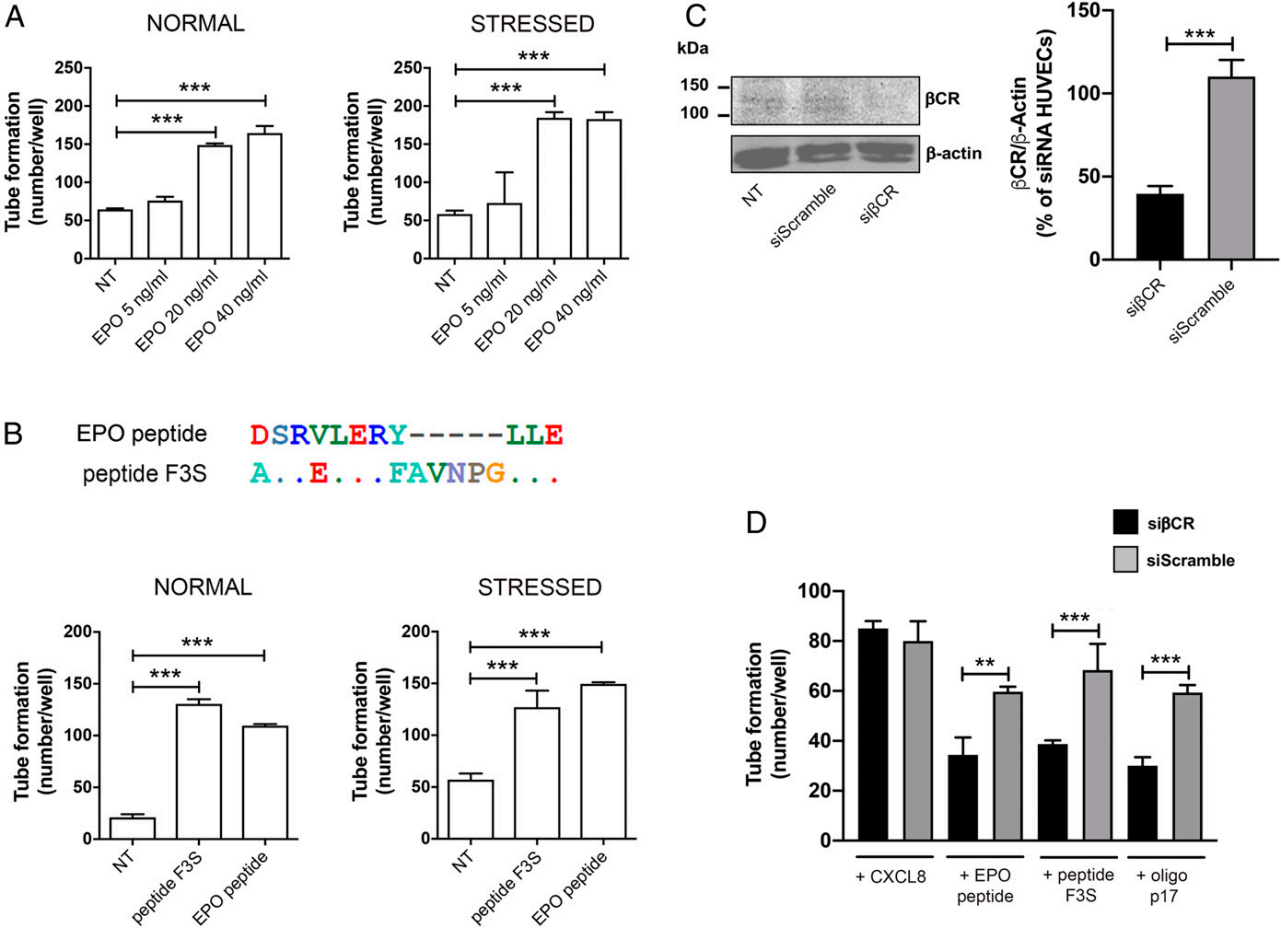
EPO peptide and peptide F3-induced angiogenesis is mediated by βCR. (*A*) HUVECs were cultured under normal (EGM containing 10% FBS) or stressed conditions (EBM containing 0.5% FBS) for 16 h at 37 °C and then stimulated for 8 h at 37 °C with 5, 20, or 40 ng/mL EPO in complete medium. NT, not treated. (*B*, *Upper*) Peptide F3 has been modeled on the region of EPO showing the maximum rate of mimicry. (*B*, *Lower*) HUVECs were cultured and stimulated as above. NT, not treated. Values reported for tube formation are the mean ± SD of one representative experiment, of three with similar results, performed in triplicate. Statistical analysis was performed by one-way ANOVA, and the Bonferroni post hoc test was used to compare data (***P < 0.001). (*C*) Western blotting analysis (*Left*) performed 72 h after nucleofection of HUVECs with βCR siRNA (siβCR) and control siRNA (siScramble). The densitometric data (*Right*) are corrected by β-actin levels and expressed as percentage of siβCR (means ± SD, *n* = 4). Statistical analysis was performed by *t* test, ***P < 0.001 (siβCR vs. siScramble). (*D*) Seventy-two hours after nucleofection with siβCR or siScramble, HUVECs were stimulated for 8 h at 37 °C with 10 ng/mL CXCL8, EPO peptide, peptide F3S, or oligomeric p17. Values reported for tube formation are the mean ± SD of one representative experiment, of three with similar results, performed in triplicate. Statistical analysis was performed by one-way ANOVA, and the Bonferroni post hoc test was used to compare data (**P < 0.01, ***P < 0.001).

Tsiakalos et al. (25) described a 63% sequence homology of the p17 area partially overlapping peptide F3 with the first 20 aa. of the EPO N-terminal region. Since EPO is known to have proangiogenic activity (26), we tested its capability to promote angiogenesis on HUVECs cultured under normal or stressed culture conditions. Similarly to peptide F3, EPO stimulated angiogenesis in both cell culture conditions in a dose-dependent manner, with optimal activity reached at 20 ng/mL (Fig. 2*A*). The observed sequence homology between p17 and EPO prompted us to construct new peptides derived from EPO (EPO peptide: aa. 8–18) and p17 (peptide F3S: aa. 37–52) (Fig. 2 *B*, *Upper*) and test them for tube-like structure formation on Matrigel. As shown in Fig. 2 *B*, *Lower*, EPO and F3S peptides promoted angiogenesis on HUVECs cultured under both normal and stressed conditions.

### The Angiogenic Activity of Oligomeric p17 Is Linked to βCR Interaction.

Since the angiogenic activity of EPO occurs through βCR signaling, following EPO binding to EPO receptor (EPOR)/βCR heterodimers (27), we checked for the involvement of βCR in the angiogenic activity of oligomeric p17. We used small interfering RNA (siRNA) to inhibit the expression of βCR (siβCR) on HUVECs. As shown in Fig. 2*C*, βCR protein expression remained strongly inhibited by the specific siβCR for 72 h following HUVEC nucleofection as compared with control—siScramble—nucleofected HUVECs (% of control HUVECs: 70.3 ± 13.0%). Inhibition of βCR expression by siβCR prevented oligomeric p17-driven angiogenesis compared to siScramble HUVECs (Fig. 2*D*). Similar results were obtained with EPO and F3S peptides. As shown in Fig. 2*D*, both peptides did not exert angiogenic activity on siβCR HUVECs, whereas they promoted angiogenesis in siScrambled HUVECs. Surface plasmon resonance (SPR) confirmed the capability of oligomeric—but not monomeric—p17 to specifically interact with βCR in the absence of any interaction with EPOR. However, EPO was found to strongly interact with the homodimeric EPOR only (*SI Appendix*, Fig. S5).

### Angiogenic Activity of SIV-Derived Peptides.

CXCR1-mediated and CXCR2-mediated angiogenesis occurs through an epitope shared between HIV-1 and HIV-2 (10, 16, 19). To understand if the capability of the epitope represented by peptide F3S to promote angiogenesis was a newly acquired function of HIV-1 during human host adaptation or preexisted in its ancestors SIVcpz and SIVgor, we synthetized two different peptides derived from SIVcpz and SIVgor matrix proteins with the closest sequence to the F3S peptide. A third peptide mimicking the same epitope expressed in the HIV-2 and SIVsmm matrix proteins (peptide HIV-2/smm) was also synthesized and tested for its angiogenetic activity. *SI Appendix*, Fig. S6*A* shows that the sequence of the F3S peptide differs only for few aa. mutations (mostly conservatives) from that of peptide cpz or peptide gor, whereas it shows consistent differences with the peptide representative of the same region on the HIV-2/smm matrix protein. As shown in *SI Appendix*, Fig. S6*B*, similarly to peptide F3S, both peptide gor and peptide cpz were able to induce angiogenesis on HUVECs cultured under normal conditions. At the same time, peptide HIV-2/smm did not show any angiogenic activity. This result excludes a gain of function of p17 during HIV-1 adaptation to the human host.

### Phylogenetic Analysis of the 37–52 p17 Fragment.

**Fig. 3.**
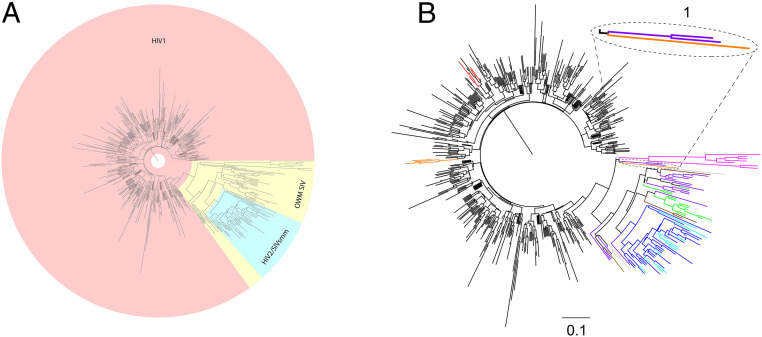
Phylogenetic tree of 37–52 p17 fragment. The tree of fragment 37–52 p17 was obtained using the representative fragment for each cluster. (*A*) The HIV-1/SIVcpz and SIVgor, OWM SIV, and HIV-2/SIVsmm branches are highlighted in pink, yellow, and azure, respectively. (*B*) The cluster of SIVcpzPts (orange) with two SIVgsn (purple) is shown in area 1. SIVcpzPts here pointed out is the SIV closely related to HIV-1, between HIV1/SIVcpz-gor and OWM SIV/HIV2-SIVsmm branches. Color code: black, HIV1; red, SIVcpzPtt and SIVgor; orange, SIVcpzPts; fuchsia, SIVcol/wrc (colobus genus); purple, SIVgsn/mon/mus/asc/deb/lst/den/syk/blu/sol (*cercopithecus* genus); green, SIVsab/tan/ver/grv/mal (*chlorocebus* genus); brown, SIVmnd/drl (*mandrillus* genus); silver, SIVrcm/agi (*cercocebus* genus); olive, SIVtal; blue, SIVsmm; azure, HIV-2.

The dataset of p17 sequences was aligned, and the 37–52 p17 fragments were selected and clustered (*SI Appendix*, Table S1). The fragment representative of 608 clusters was used to perform a phylogenetic analysis using the neighbor-joining (NJ) method (28, 29). The tree shows a well-defined grouping in three branches (Fig. 3*A*): 1) HIV-1/SIVcpz-gor branch includes HIV-1, SIVcpz, the subspecies *Pan troglodytes troglodytes* (cpzPtt) and *Pan troglodytes schweinfurthii* (cpzPts), and SIVgor. However, one SIVpts changed its topological position (Fig. 3*B*), becoming a sister group of this branch clustering with SIV *greater spot-nosed monkey* (SIVgsn) in the Old World Monkey (OWM) SIV branch; 2) HIV-2/SIVsmm branch includes HIV-2 and SIVsmm strains only; 3) OWM SIV branch groups almost together. SIV *colobus monkey* (col) and SIV *western red colobus* (SIVwrc) are located distantly from the others OWM SIV, showing divergent aa. sequences if compared to all the others (30, 31).

To validate the accuracy of our study, another widely used method that makes different statistical assumptions with respect to NJ, namely maximum likelihood (ML), (32, 33) was used. The tree obtained shows a high degree of similarity confirming the same evolutionary relationship between SIVs and HIVs (*SI Appendix*, Fig. S7). The phylogenetic study highlights that the active p17 fragment is unique and specific for HIV-1, likewise, the inactive one is for HIV-2, according to biological data.

### Active Moiety of the 37–52 p17 Fragment.

To identify the active moiety inside 37–52 p17 fragment, three subfragments (S1, S2, and S3) were designed by sequence and structural alignment of HIVs and EPO fragments and descriptive statistics of 37–52 p17 fragment sequences dataset. Subsequently, the subfragments were associated with the biological function of the 37–52 p17 fragment through further phylogenetic analysis. HIVs S1 subfragments (aa. 37–44) have residues with biochemical properties similar to EPO 8–15 residues, HIVs S2 subfragments (aa. 45–49) are insertions if compared to the EPO 8–18 fragment, and HIVs S3 subfragments (aa. 50–52) are highly conserved with respect to the EPO 16–18 sequence (*SI Appendix*, Fig. S8*A*).

From the comparative structural analysis of fragments, the secondary elements observed in X-ray and NMR structures were mapped above sequence fragments by structural sequence alignment. The helix 2 of HIVs, corresponding to 37–44 ASRELERF (HIV-1) and AANELDRF (HIV-2) S1 subfragments, aligns to the erythropoietin αA helix, corresponding to the 8–15 DSRVLERY subfragment (*SI Appendix*, Fig. S8*B*). Corresponding regions of HIVs S2 and S3 subfragments show a different helix propensity. In particular, the folding of these regions in HIV-1 is dependent on the p17 structural environment as indicated by comparative studies of NMR, X-ray, and molecular dynamics (34). Folding of the same regions in HIV-2 is environment-dependent and limited to a few residues only. Therefore, folding of the S2 and S3 subfragments could influence the biological activity of the 37–52 fragment due to their structural environmental dependence.

**Fig. 4.**
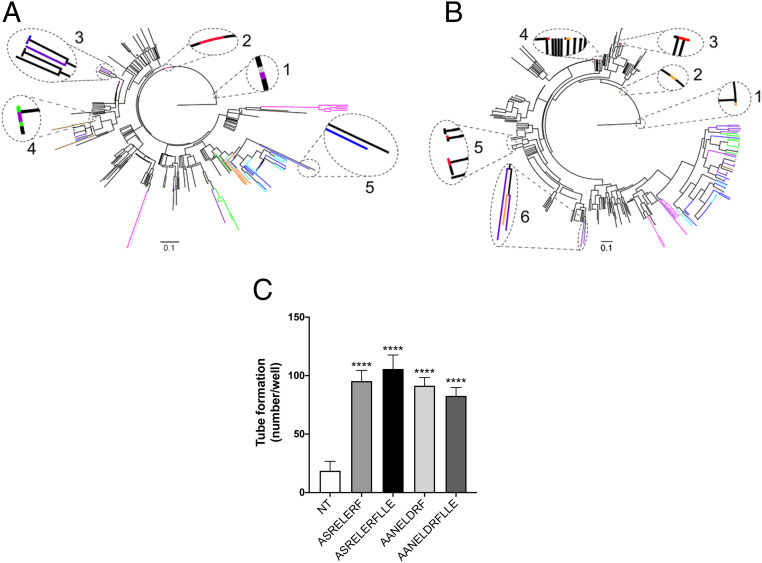
Identification of the p17 functional epitope for angiogenesis. (*A*) The phylogenetic tree of S1 subfragment shows a nonhomogeneous assembling of viruses. Areas 1–6: highlighted some branches and clades of the tree; different OWM SIV and SIVsmm groups with HIV-1. Area 1: HIV-1 (black), SIV *cercopithecus* (purple), SIV *cercocebus* (gray). Area 2: HIV-1 (black), SIVcpz/gor (red). Area 3: HIV-1 (black), SIV *cercopithecus* (purple), SIVsmm (blue). Area 4: HIV-1 (black), SIV *cercopithecus* (purple), SIV chlorocebus (green). Area 5: HIV-1 (black), SIVsmm (blue). Area 6: HIV-1 (black), SIVsmm (blue), SIVcpzPtt (orange), HIV-2 (azure). (*B*) The phylogenetic tree of S2 subfragment shows the typical evolutionary path of HIV-1/SIVs. Areas 1–5 highlight SIVcpz and SIVgor grouping together with HIV-1. Area 6 highlights the clade formed by SIVcpzPts and two SIVgsn grouping with HIV-1. Areas 1–2: HIV-1 (black), SIVcpzPts (orange). Areas 3–5: HIV-1 (black), SIVcpz/gor (red). Area 4: HIV-1 (black), SIVcpz/gor (red), SIVcpzPts (orange). Area 6: SIVcpzPts (orange), SIV *cercopithecus* (purple). Color code: black, HIV-1 and HIV-1/SIVcpz-gor; red, SIVcpzPtt and SIVgor; orange, SIVcpzPts; blue, SIVsmm; azure, HIV-2; purple, SIVgsn/mon/mus/asc/deb/lst/den/syk/blu/sol (*cercopithecus* genus); green, SIVsab/tan/ver/grv/mal (*chlorocebus* genus); brown, SIVmnd/drl (*mandrillus* genus); silver, SIVrcm/agi (*cercocebus* genus); olive, SIVtal; fuchsia, SIVcol and SIVwrc (*piliocolobus/colobus* genus). (*C*) HUVECs were cultured under normal condition and then stimulated for 8 h at 37 °C with 10 ng/mL peptide ASRELERF, peptide ASRELERFLLE, peptide AANELDRF, or peptide AANELDRFLLE. NT, not treated. Values reported for tube formation are the mean ± SD of one representative experiment, of three with similar results, performed in triplicate. Statistical analysis was performed by one-way ANOVA and the Bonferroni post hoc test was used to compare data (****P < 0.0001).

The descriptive statistics of S1, S2, and S3 (*SI Appendix*, Table S2) allowed us to hypothesize that the S3 subfragment is not related to the function of peptides, due to its uniform distribution over all sequences. In order to study if S1 and S2 subfragments were related to the biological function, two different phylogenetic analyses were carried out starting from the cluster dataset of 37–52 p17 fragments. Analyses of the trees showed that the S1 subfragment tree has not defined branches and does not show a typical HIV-1/SIV evolutionary path (Fig. 4*A*). However, the evolution of S2 corresponds to the previously analyzed 37–52 fragment, excluding the clade formed by SIVcpzPts (cluster 314) and the two SIVgsn that in S2 assembles with HIV-1 (Fig. 4*B*). The two different ensembles for S1 and S2 were confirmed by the ML method (*SI Appendix*, Figs. S9 and S10). These results suggest that the S2 fragment is the evolutionary switch component that drives the biological activity of the 37–52 fragment.

### Angiogenic Activity of an F3-Derived 8 aa.-Long Peptide.

Difference between F3S and EPO peptides consists in the presence of a 5-aa. insertion (AVNPG) in the peptide of viral origin. In order to test the role of this aa. insertion in the peptide’s angiogenic activity, we synthesized a 11-aa.-long peptide devoid of the AVNPG sequence (ASRELERFLLE). At the same time, we tested the activity of a 8-aa.-long peptide, reproducing the first aa. sequence of the F3S peptide (ASRELERF). As shown in Fig. 4*C*, both peptides were able to significantly promote angiogenesis on HUVECs. This result attests that only 8 aa. are sufficient to sustain the angiogenic activity mediated by the F3S peptide, thus supporting data obtained by comparative structural analysis of fragments. Then, we evaluated the possibility that also peptides derived from peptide HIV-2/smm lacking the GLAES insertion may be endowed of angiogenic activity. For this reason, we synthesized two (11- and 8-aa.-long) peptides containing or not the LLE aa. stretch (AANELDRFLLE and AANELDRF). As shown in Fig. 4*C*, these two peptides resulted endowed of potent angiogenic activity.

## Discussion

The work reported here shows that both monomeric and oligomeric p17 promote angiogenesis and lymphangiogenesis at nanomolar concentrations. This was found to occur because of the presence in the viral protein of two distinct functional epitopes. A first epitope (aa. 17–37) working under stress conditions only that interacts with CXCR1 and CXCR2; the second (aa. 37–44) acting also under normal culture condition was found to act through βCR activation. Biologically active p17 is secreted by HIV-1–infected cells even in the absence of an active viral protease (7). It is present in serum of patients at nanomolar concentrations (35) and is also detected in different tissues and organs, where it accumulates and persists for years even in patients undergoing successful cART (16, 36). Therefore, p17-driven angiogenesis and lymphangiogenesis may likely occur in the HIV-1–infected host. Angiogenesis and lymphangiogenesis are essential in supporting proliferation and survival of lymphomas, the most common malignancy in the AIDS population even in the cART era (37). Altogether, these findings call for the development of new preventive and/or treatment strategies aimed to hamper the p17-mediated EC stimulation.

Here, we also show by SPR that oligomeric p17 is able to directly engage βCR and promote a βCR-mediated potent angiogenic activity on human ECs. To our knowledge, oligomeric p17 is the only protein able to directly bind and activate βCR. Oligomerization is a prerequisite for p17 to bind to βCR. The known capability of p17 to interact with heparan sulfate proteoglycans (14) makes possible—as for many heparin-binding chemokines—a better presentation of p17 to βCR, by setting up synergistic and cooperative interactions leading to increased concentrations and, consequently, oligomerization of the viral protein at the EC surface.

Previous reports have demonstrated that within the heterodimeric EPOR/βCR complex, βCR is the one responsible for the EPO angiogenic activity (38). Up to date, the epitope responsible for the angiogenic activity of EPO was still unknown. Because of antigenic mimicry, by identifying the angiogenic epitope of p17 linked to βCR activation, we also uncovered the epitope on EPO responsible for βCR-mediated angiogenic activity. It was found to reside at the N-terminal region of the human protein, spanning from aa. 8 to 18. This 11-aa.-long EPO peptide was found per se to promote angiogenesis. Data obtained by SPR clearly showed the capability of EPO to interact with the homodimeric EPOR but not with the homodimeric βCR. βCR forms heterodimers with the alpha chain receptor of the hematopoietin receptor superfamily, whose members belong to IL-3, IL-5, and granulocyte-macrophage colony-stimulating factor (GM-CSF) receptors. In addition to its importance in stabilizing the binding of ligands to their respective receptors, βCR is the principal signal transducing subunit within the alpha/beta receptor complex (38). In this respect, our data on angiogenesis suggest that in the context of the EPOR/βCR heterodimers expressed on ECs, the βCR-activating angiogenic epitope becomes functional following EPO interaction with EPOR.

HIV-1 infection and replication are strongly related to inflammation, cell activation, and differentiation status. During HIV-1 infection, even in ART-treated virally suppressed individuals, parameters of immune activation and inflammation remain persistently elevated and are predictors of a series of clinical conditions collectively known as serious non-AIDS events (39). However, HIV-1 controllers have low inflammation and a low level of inflammatory cytokines (40). HIV-2 infection is generally considered a naturally occurring form of attenuated HIV-1 disease, characterized by slow CD4^+^ T-cell decline, undetectable-to-low levels of circulating virus, and limited impact on the mortality of infected adults (41). Moreover, the rate of increase in immune activation is much lower in HIV-2 than in HIV-1 disease (42), whereas levels of proinflammatory cytokines did not increase during HIV-2 infection (43). These findings attest for a strong pathogenetic difference between HIV-1 and HIV-2.

The origin of HIV-1 and HIV-2 has been intensively studied since the appearance of AIDS pandemia. Previous data have highlighted how HIV-1 ancestors were already capable of breaking into and productively infect human cells (1). Subsequently, the HIV-1 pandemic group M accounting for >98% of all human infections (44) acquired a specific antihuman tetherin function to escape from this host restriction factor, by mastering a switch from Nef-mediated to Vpu-mediated tetherin antagonism (45). This did not occur in HIV-2 or in the HIV-1 not pandemic groups N, O, and P. Moreover, an adaptive change at position 30 of p17 emerged as a major determinant of human-specific adaptation (20, 21). Here, we show that an epitope expressed on p17 responsible for the βCR-mediated angiogenesis is also present in the matrix protein of SIVcpz and SIVgor but not in that of HIV-2 and its ancestor SIVsmm. This finding prompted us to evaluate the evolutionary trajectory of the βCR-activating epitope. According to biological data, evolution of the epitope responsible for angiogenic activity, spanning from aa. 37–52, shows a clear differentiation between HIV-1/SIVcpz-gor and HIV-2/SIVsmm branches, thus highlighting this epitope on p17 as a clear divergent signature discriminating HIV-1 and HIV-2 ancestors. It is therefore likely that the p17 region spanning from aa. 37 to 52 on p17 is constrained to allow functional epitopes to perform unavoidable interactions.

Comparative structural analysis of HIVs and SIVs fragments with the EPO one, together with biological data, showed that an active EPO-like moiety at position 37–44 was present in the matrix protein of all viruses. Indeed, the distribution of this subfragment among the phylogenetic tree did not show a typical HIV-1/SIV evolutionary path as for the entire 37–52 fragment. However, a 5-aa.-long insertion at position 45–49 was found to be responsible for the switch-on/off of the angiogenic activity. The switch-on insertion was mostly represented by the aa. stretch AV[L]NPG on the matrix protein of HIV-1 and its ancestors, whereas the switch-off insertion was represented by the aa. stretch GLAES for HIV-2 and its ancestors. Interestingly, the evolution of the 5-aa.-long sub fragment matched to that traced by the entire 37–52 fragment, leading us to conclude that the evolutionary switch component for the βCR-dependent angiogenic activity resides in the 45–49 aa. stretch. Computational analysis further revealed that folding of the 45–49 subfragments can influence the biological activity of the entire epitope due to its structural environmental dependence.

**Fig. 5.**
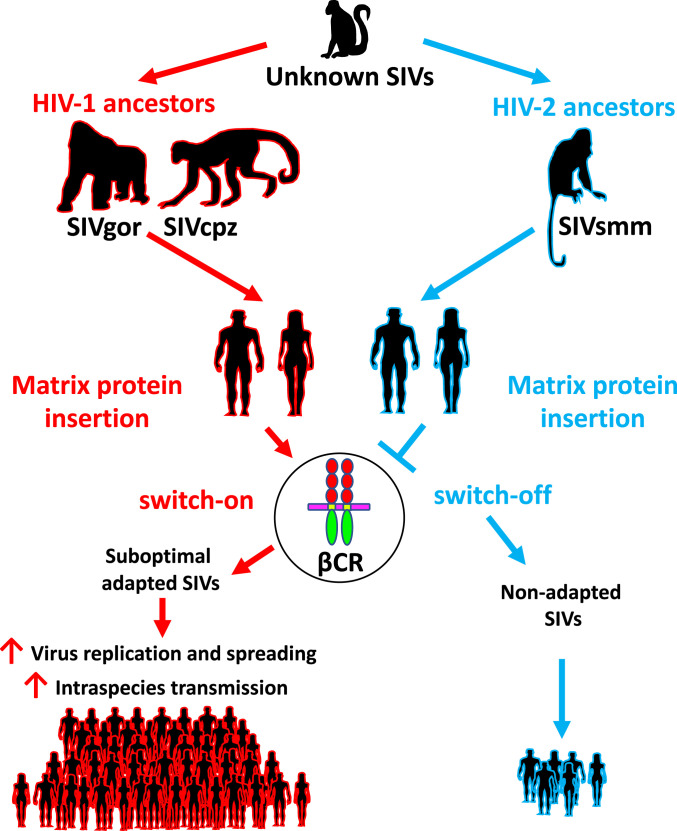
Hypothesis for the role of βCR in SIV suboptimal adaptation to the human host.

Based on this evidence, it is tempting to speculate on the meaning of the appearance of the βCR-binding epitope on the matrix protein of the HIV-1 ancestors. βCR is known to activate three different signaling pathways (46, 47) and to regulate a variety of cellular processes including proliferation and differentiation. Cytokines belonging to the βCR family regulate the survival and function of monocytes, macrophages, and dendritic cells. Moreover, they promote leukocyte differentiation and coordinate an inflammatory infiltration of lymphoid cells and CD4^+^ T cell proliferation (48). Similarly, p17 has been shown to act as a virokine able to promote a proinflammatory microenvironment, attract HIV-1 target cells to the site of viral infection, and enhance HIV-1 replication (8, 11, 22). Although the current study design does not allow causality to be inferred, it does support the hypothesis that p17/βCR interaction may play a crucial role in chemoattracting HIV-1 target cells to the site of viral infection and favoring HIV-1 replication and spreading. Therefore, acquisition of an epitope on the matrix proteins of HIV-1 ancestors capable of triggering βCR may have represented a critical step to enhance viral aggressiveness and early human-to-human SIVcpz/gor dissemination (Fig. 5), before additional genetic acquisitions and/or mutations could subsequently accumulate to facilitate virus adaptation to the new host.

In conclusion, our data strengthen the key role of p17 in the evolutionary trajectory of HIV-1. Recently, βCR activation by different ligands was shown to impact on multiple cell types, organs, and biological systems, thereby controlling the balance between health and disease (49). The hypothesis that p17/βCR interaction and βCR abnormal stimulation may also play a role in sustaining chronic activation and inflammation, thus marking the pathogenic difference between HIV-1 and HIV-2, needs further investigation. Finally, as a direct βCR ligand, oligomeric p17 and/or the βCR-activating peptides identified here may prove useful to uncover the βCR active site and study the potential risks and benefits of its stimulation.

## Materials and Methods

For a complete description of the source of materials and methods, see *SI Appendix*, *Materials and Methods*.

It includes description of recombinant monomeric and oligomeric LPS-free HIV p17 protein production, cell culture information, and silencing procedures. It also includes description of the following assays: tube like-structures, wound healing, aortic ring, CAM, Western blotting, SPR as well as data collection, alignment, phylogenetic, and statistical analysis.

## Supplementary Material

Supplementary File

## Data Availability

All study data are included in the article and supporting information.
