# Supplementary material for: Evolution toward beta common chain receptor usage links the matrix proteins of HIV-1 and its ancestors to human erythropoietin
Source: Proc Natl Acad Sci U S A. 2020 Dec 28;118(2):e2021366118. doi: 10.1073/pnas.2021366118 (PMC7812818; doi:10.1073/pnas.2021366118)
Supplement: Supplementary File [file pnas.2021366118.sapp.pdf]

Supporting Information

Materials and Methods

Recombinant proteins

Purified endotoxin (lipopolysaccharide)-free recombinant monomeric p17 (from clone BH10 of clade B isolate), glutathione S-transferase (GST), and the HIV-1 capsid protein p24 (p24) were produced as previously described (9). The absence of endotoxin contamination (< 0.25 endotoxin units/ml) in protein preparations was assessed by the Limulus amebocyte assay (Associates of Cape Cod, Inc.). In some experiments we used aggregated p17 to stimulate ECs. Aggregation of p17 was achieved as previously described (25).

Cell Cultures

ECs were isolated and characterized as previously described (17) and cultured in endothelial growth medium (EGM) (Lonza, Milan, Italy) containing 10% (vol/vol) fetal bovine serum (FBS) . Human aortic endothelial cells (HAECs) were purchased from Clonetics (San Diego, CA, USA) and in EGM-2MV (Lonza) containing 10% (vol/vol) (FBS). Human lung microvascular endothelial cells (HMVEC-Ls) were purchased from Clonetics (San Diego) and cultured in EGM-2MV (Lonza) containing 10% (FBS). Human primary lymph node-derived endothelial cells (LN-LECs) have been developed and characterized as previously described (23). Cells were cultured in endothelial growth medium (EGM) (Lonza) containing 10% and supplemented with VEGF-C (25 ng/mL) (Reliatech, Wolfenbuettel, Germany). All experiments were carried out with cells at passages 2-6.

Silencing by siRNA technique

Nucleoporation of HUVECs was performed using the Amaxa Nucleofector Technology (Lonza) following the manufacturer's protocol. Small interfering RNAs (siRNAs) were added to 1 x 10^6 cells resuspended in 100 µl of nucleofection buffer. Silencing was carried out using 300 nM of Beclin-1

siRNA (Cell Signaling Technology, Boston, MA) or 10 μM CD131 siRNA (Santa Cruz Biotechnology). Irrelevant siRNAs (siScramble; Cell Signaling Technology) were used as a negative control.

In vitro tube-like structure formation assay

ECs were grown under normal (10% FBS) or stressed (serum starved; 0.5% FBS) conditions for 16 h in endothelial basal medium (EBM) and then harvested and resuspended in EGM containing 10% FBS. Cells were seeded (5 x 10^4 per well) in wells coated with growth factor-reduced Cultrex basement membrane extract (BME; Trevigen Gaithersburg, MD) and left untreated or treated with 10 ng/ml of GST, p24, p17, Erythropoietin (EPO), CXCL-8 (R&D), different p17 derived peptides or EPO peptide. When indicated, cells were nucleofected with siRNAs specific for Beclin-1, βCR or with siScramble. In some experiments, ECs were pretreated for 16 h at 37°C with 5 mM 3-methyladenine (3-MA) (Sigma-Aldrich, St. Louis, MO). When reported starved ECs were pretreated with 2.5 μg/ml of MAb to CXCR1 (MAb 330; R&D, Minneapolis, MN) and CXCR2 (MAb 331; R&D) alone or in combination (1.25 μg/ml each) or with an isotype-matched MAb (2.5 μg/ml; R&D) for 1 h at 37°C before p17 stimulation. Wells were then analyzed for the formation of tube structures.

Wound-healing assay

ECs were plated on 24-well plates (1 x 10^5 cells per well) in EGM containing 10% FBS. Confluent monolayers were nutrient starved for 16 h in the presence or absence of 3-MA (5 mM) (Sigma-Aldrich) and then scratched using a 200 μl pipette tip. After being washed, cells were left untreated or treated with 10 ng/ml of GST, p24, p17, Peptide F2 or Peptide F3.

Aortic Ring Assay

The rat aortic ring assay was performed as previously described (17). Briefly, the dorsal aorta was excised from 6-wk-old Sprague-Dawley rats. Rings, 1-mm thick, were embedded in collagen gel and

then incubated with serum-free EBM containing peptide F2, peptide F3 (10 ng/ml), or p17 (20 ng/ml) (Thermo Fisher Scientific, Rodano, Italy). The plates were incubated for 10 days and angiogenesis was quantified by counting the number of microvessels originating from aortic rings.

Chick Chorioallantoic (CAM) Assay

Fertilized White Leghorn chicken eggs (30 per group) were incubated at 37 °C at constant humidity. On day 3, a square window was opened in the shell, and 2-3 ml of albumin was removed to allow detachment of the developing CAM. The window was sealed with a glass, and the eggs were returned to the incubator. On day 8, eggs were treated with 1 mm^3 sterilized gelatin sponges (Gelfoam; Upjohn, Kalamazoo, Mich, MI) placed on top of the growing CAM, as previously described (17) and loaded with 1 μl of PBS (negative control), 1 μl of PBS containing 200 ng of p17, 50 ng of peptide F2 or peptide F3. CAMs were examined daily until day 12 and photographed in ovo with a stereomicroscope equipped with a camera and image analyzer system (Olympus). At day 12, the angiogenic response was evaluated by the image analyzer system and counted as the number of vessels converging toward the sponge.

Western blotting

Human umbilical vein endothelial cells (HUVECs) lysates were loaded for electrophoresis on a 10% polyacrylamide gel, and transferred onto PVDF membranes. Membranes were then blocked and probed overnight at 4° C with primary mouse anti-CD131 (1C1, 1:200; Santa Cruz Biotechnology, Dallas, Texas, USA) and mouse anti-β-Actin (C4) (Santa Cruz Biotechnologies) antibodies. Membranes were washed and incubated with anti-mouse peroxidase-conjugated secondary antibody (1:3000; Cell Signaling Technology, Danvers, MA, USA). Antibody detection was accomplished with the ECL system (Euroclone S.p.a., Milan, Italy), and protein bands were quantified with the ImageJ analysis system. Band densities were represented as an index of CD131 corrected by β-Actin.

SPR Binding Assay

SPR measurements were performed on a BIAcore X100 instrument (GE Healthcare, Chicago, Illinois, USA). For the study of Erythropoietin receptor (EPO-R) interactions, homodimeric recombinant EPOR (Recombinant human EPOR protein Fc chimera) was captured onto a CM5 sensorchip containing pre-immobilized protein A (Merck KGaA, Darmstadt, Germany), allowing the immobilization of 3500 resonance units (RU), equal to 63 fmol/mm^2 of EPOR. A sensorchip precoated with protein A alone was used to evaluate nonspecific binding and for blank subtraction. EPO, monomeric or oligomeric p17 (300 nM) in 10 mM HEPES, pH 7.4 containing 150 mM NaCl, 3 mM EDTA, and 0.05% surfactant P20 (HBS-EP$^+$) were injected over EPOR (Abcam, Cambridge, UK), or protein A surfaces for 4 min and then washed until dissociation. After each run, the sensorchip was regenerated by injection of 2.0 mM NaCl in HBS-EP$^+$. For the study of βCR interactions, anti-His antibody (anti His-Tag Rabbit Polyclonal Antibody, Origene, Rockville, MD, USA) was immobilized onto a CM5 sensorchip using standard amine-coupling chemistry as previously described (20). Then, recombinant homodimeric βCR with a C-terminal 6-His tag (50 μg/ml) (Recombinant Human Common β Chain His Tagged (CD131/βCR) (R&D Systems, Minneapolis, USA) was injected over the anti-His surface, allowing the immobilization of 156 RU, equal to 1,2 fmol/mm^2 of the receptor. A sensorchip coated with anti-His antibody was used as a negative control and for blank subtraction. EPO, monomeric or oligomeric p17 (300 nM) in HBS-EP$^+$ were injected over βCR or anti-His surfaces for 4 min and then washed until dissociation. After each run, the sensorchip was regenerated by injection of 2.0 mM NaCl in HBS-EP$^+$.

Data collection, alignment and phylogenetic analysis

Several steps were involved in the bioinformatics study:

1) A dataset of 33220 sequences of p17 was retrieved from Uniprot.

2) A unique alignment has been generated using Clustal Omega and Muscle and manually curated discarding sequences with missing, unknown (X) or uncertain (B and Z) residues.
3) The remaining 33180 sequences were clustered based on variability of spanning residues 37-52, in 608 clusters. Over 33180 sequences, 32753 are HIV-1, 39 are HIV-2 and 388 are SIVs: 31 SIVcpz, 9 SIVgor, 239 SIVsmm and 109 old world monkey (OWM) SIV. The dataset includes a considerable proportion of HIV-1 sequences in order to identify all mutational events that occurred during 37-52 p17 fragment phylogeny.
4) The 37-52 p17 fragment sequences of the 608 clusters were aligned using the software Muscle.
5) For each of the three selected fragments we have performed the following procedure:

Trees 37-52 p17 fragment, S1 and S2 were obtained using the sequences of fragments 37-52, 37-44 and 45-49 respectively. Trees have been generated using the neighbour-joining (NJ) and a maximum likelihood (ML) methods. For the NJ analysis we used Clustal W with default setting and bootstrap of 1000; for ML we used PhyML program (v.3.0) with the generalised time reversible substitution model and subtree pruning and regrafting branch swapping and Raxml with similar settings. Bootstrap support values were calculated using > 1,000 pseudo-replicate trees. The best-fitting model of nucleotide substitution was determined using MEGA (v.5). The structural study of the fragments (37-52 p17, S1 and S2) was obtained using the structures of the p17 protein of HIV-1 (pdb code: 2HMX, 1UPH, 1L6N, 1TAM and 1HIW) and HIV-2 (pdb code: 2K4H and 2k4E) using the align command of Pymol.

Statistical analysis

Data obtained from multiple independent experiments are expressed as the means ± the standard deviation (SD). The data were analyzed for statistical significance using t-test or one-way analysis of

variance (ANOVA). Bonferroni's post-test was used to compare data. Differences were considered significant at a P value of <0.05. Statistical tests were performed using Prism 8 software (GraphPad).

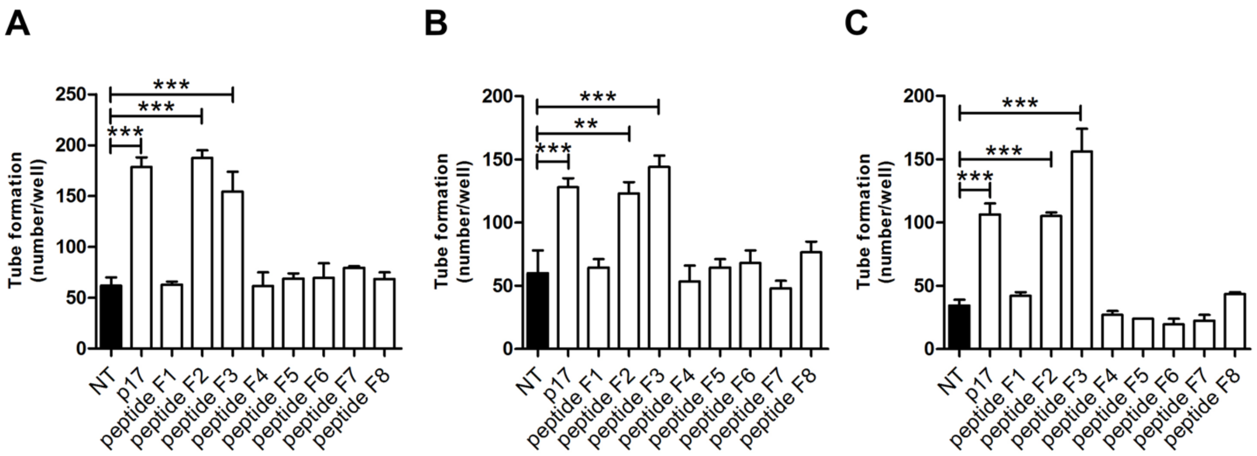

Figure S1. Peptides F2 and F3 induce angiogenesis in human ECs derived from different organs.

Human aortic endothelial cells (HAECs) (A), Human lung microvascular endothelial cells (HMVEC-Ls) (B) and Human primary lymph node-derived endothelial cells (LN-LECs) (C) were cultured under stressed condition (EBM containing 0.5% FBS) for 16 h at 37°C and then stimulated for 8 h at 37°C with 10 ng/ml of p17 or each p17-derived peptide (F1, F2, F3, F4, F5, F6, F7, F8). NT, not treated. Values reported for tube formation are the mean ± SD of one representative experiment, out of three with similar results, performed in triplicate. Statistical analysis was performed by one-way ANOVA, and the Bonferroni post-test was used to compare data (**P<0.01; ***P<0.001).

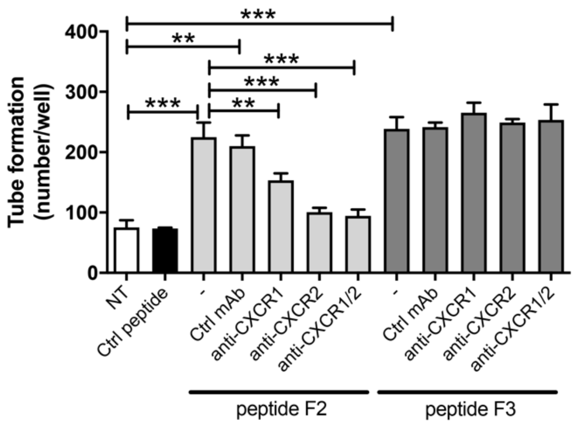

Figure S2. Peptide F2 – but not F3 – induced tube-like structure formation occurs through CXCR1 and CXCR2. HUVECs were cultured under stressed condition (EBM containing 0.5% FBS) for 16 h at 37°C and then preincubated for 1 h at 37°C with 2.5 µg/ml of a control isotype-matched mAb (Ctrl mAb), a neutralizing mAb to CXCR1 and/or a neutralizing mAb to CXCR2 and then stimulated with 10 ng/ml of peptides F2 or F3 for 8 h at 37°C. Values reported for tube formation are the mean ± SD of one representative experiment, out of three with similar results, performed in triplicate. Statistical analysis was performed by one-way ANOVA, and the Bonferroni post-test was used to compare data (**P<0.01, ***P<0.001). NT, not treated.

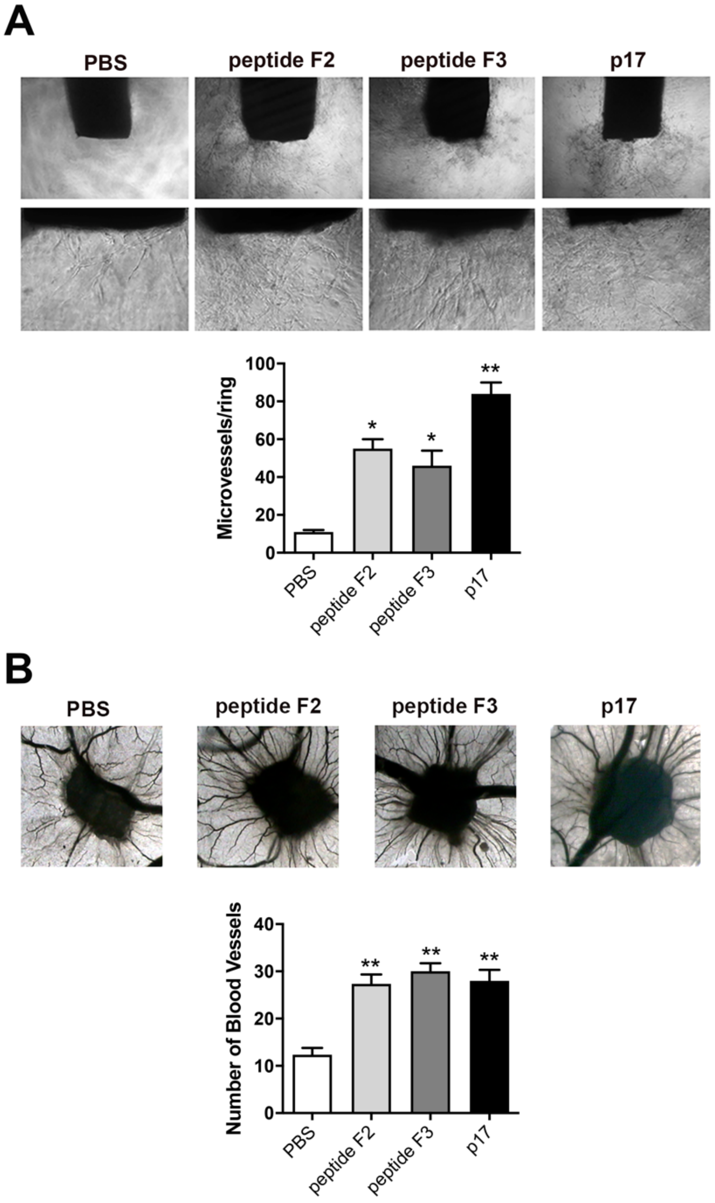

Figure S3. Peptides F2 and F3 promote vasculogenesis in rat aortic ring and CAM assays. (A) Rat aortic rings were embedded in collagen gel and incubated for 10 days in EBM containing 10 ng/ml of peptide F2 or F3. Control rings were incubated for 10 days in EBM in the presence of PBS or p17 (20 ng/ml). Microvessel structures were observed by phase microscopy on day 7. (B) Macroscopic pictures of CAMs at day 12 of incubation. Gelatin sponges were adsorbed with vehicle alone (PBS), with 200 ng of p17 or with 50 ng of peptide F2 or F3. Pictures are representative of three independent experiments with similar results. Original magnification, ×4. Data are the mean ± SD of three independent experiments. Statistical analysis was performed by one-way ANOVA, and the Bonferroni post-test was used to compare data (*P<0.05; **P<0.01).

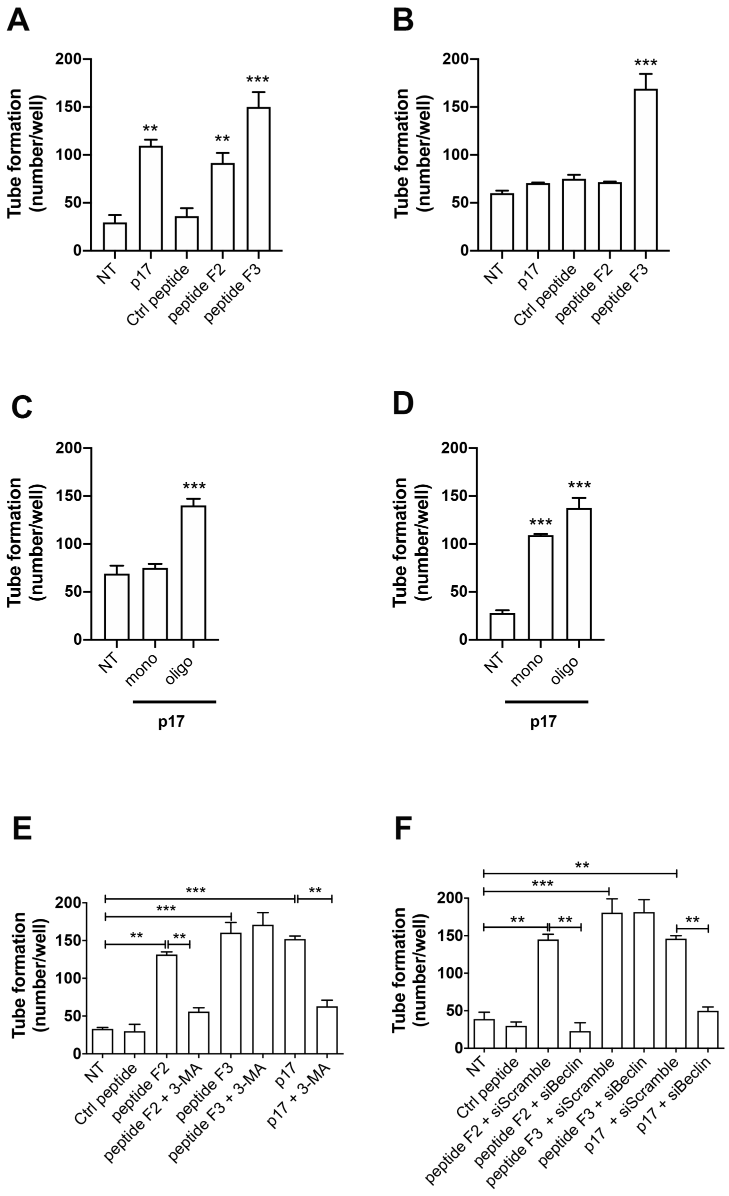

Figure S4. Angiogenic activity of p17 and p17-derived peptides upon normal and stressed culture conditions. (A) HUVECs were cultured under stressed condition (EBM containing 0.5% FBS) for 16 h at 37°C and then stimulated with 10 ng/ml of p17, peptide F2 or peptide F3 for 8 h at 37°C in complete medium. NT, not treated. (B) HUVECs were cultured under normal condition (EGM containing 10% FBS) and then stimulated with 10 ng/ml of p17, peptide F2 or peptide F3 for 8 h at 37°C. NT, not treated. (C) HUVECs were cultured under normal condition and then stimulated with 10 ng/ml of monomeric (mono) or oligomeric p17 (oligo) for 8 h at 37°C. NT, not treated. (D) HUVECs were cultured under stressed condition for 16 h at 37°C and then stimulated with 10 ng/ml of mono or oligo p17 for 8 h at 37°C in complete medium. NT, not treated. (E) HUVECs were

cultured under stressed condition for 16 h at 37°C in the presence or absence of 3-MA (5 mM) and then stimulated with 10 ng/ml of p17, peptide F2 or peptide F3 for 8 h at 37°C in complete medium. NT, not treated. (F) HUVECs were nucleofected with Beclin-1 siRNAs (siBeclin) or with irrelevant siRNAs (siScramble). Twenty-four h after nucleofection, cells were serum starved for 16 h and then stimulated with 10 ng/ml of p17, peptide F2 or peptide F3 for 8 h at 37°C in complete medium. NT, not treated. Values reported for tube formation are the mean ± SD of one representative experiment, out of three with similar results, performed in triplicate. Statistical analysis was performed by one-way ANOVA and the Bonferroni post-test was used to compare data (**$P<0.01$; ***$P<0.001$).

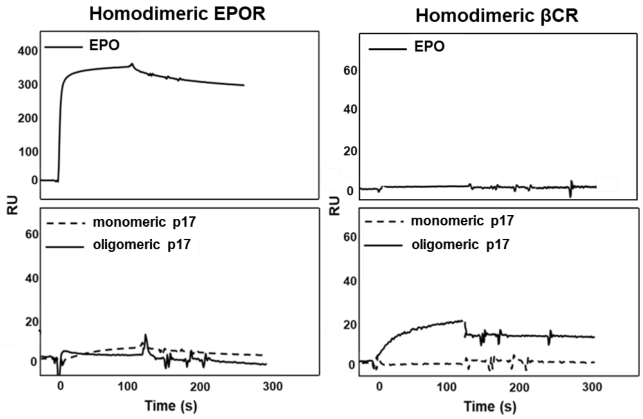

Figure S5. SPR analysis of EPO and p17 binding to homodimeric EPOR and βCR. The interaction between EPO, monomeric and oligomeric p17 with homodimeric EPOR or homodimeric βCR, were evaluated by SPR analysis. Sensorgrams report the binding of EPO (300 nM) and monomeric or oligomeric p17 (300 nM) to sensorchip coated with homodimeric EPOR (left panel) or βCR (right panel). The response was recorded in RU as a function of time.

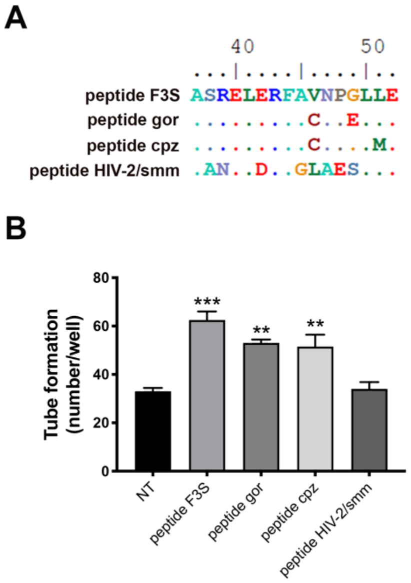

Figure S6. Angiogenic activity of peptide F3-like SIV-derived peptides. (A) Amino acid sequence of different peptides derived from the matrix protein of HIV-1 (peptide F3), SIVgor (peptide gor), SIVcpz (peptide cpz) or HIV-2/SIVsmm (peptide HIV-2/smm). (B) HUVECs were cultured under normal condition (EGM containing 10% FBS) and then stimulated with 10 ng/ml of peptides F3, gor, cpz or HIV-2/smm for 8 h at 37°C. NT, not treated. Values reported for tube formation are the mean ± SD of one representative experiment, out of three with similar results, performed in triplicate. Statistical analysis was performed by one-way ANOVA and the Bonferroni post-test was used to compare data (**P<0.01; ***P<0.001).

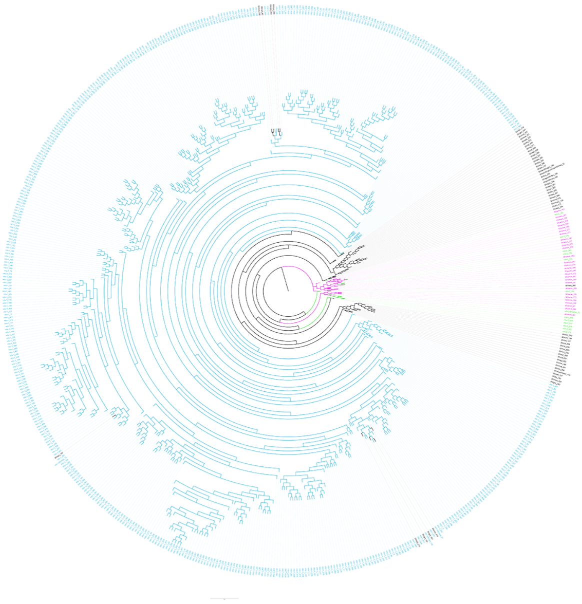

Figure S7. Tree comparison for the 37-52 p17 fragments. Phylogenetic tree of 37-52 p17 fragments constructed using the maximum likelihood (ML) method. The tree shows a comparable grouping for HIV-1, HIV-2 and SIVs. Viruses color code: HIV-1 azure, HIV-2 green, SIVsmm purple (and other SIVs in black).

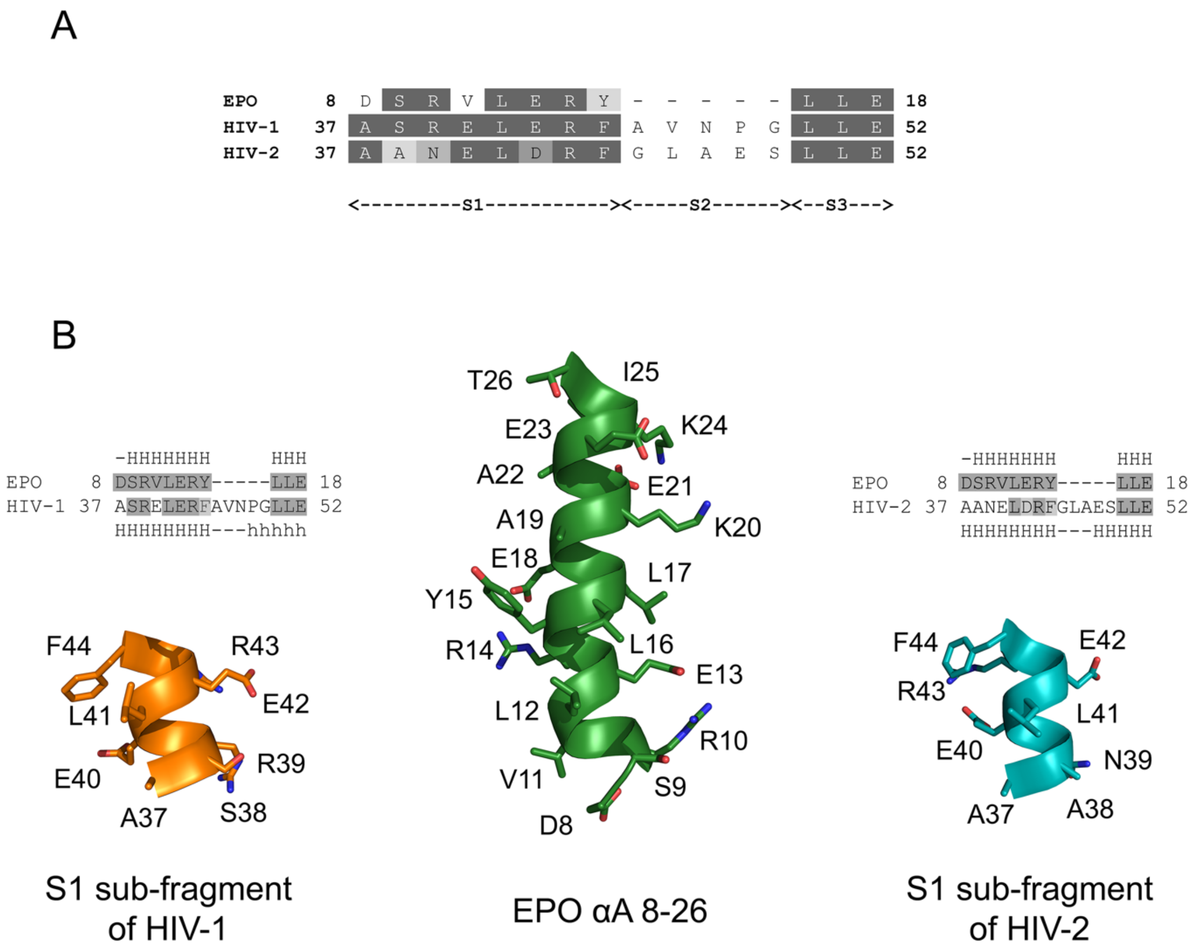

Figure S8. Alignment and structural analysis of fragments. (A) Alignment of 37-52 HIV-1 and 8-18 EPO fragments. Biochemical amino acid similarities between EPO and HIV-1 are shown in gray gradient. (B) Structural comparison between S1 of HIV-1 fragments, with αA helix of EPO. In the alignment H correspond to α-helix, while h to a α-helix propensity. Biochemical amino acid similarities between EPO and HIV-1 are shown in gray gradient.

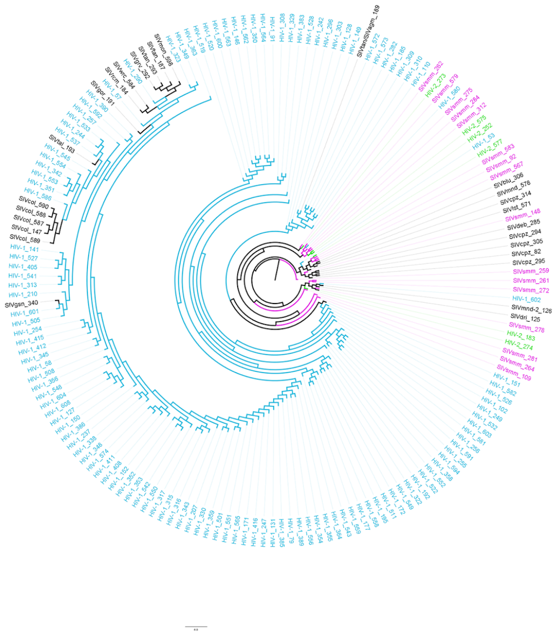

Figure S9. S1 tree comparison. Phylogenetic tree of S1 constructed using the maximum likelihood (ML) method. The tree shows a comparable grouping for HIV-1, HIV-2, SIVs. Viruses color code: HIV-1 azure, HIV-2 green, SIVsmm purple (and other SIVs in black).

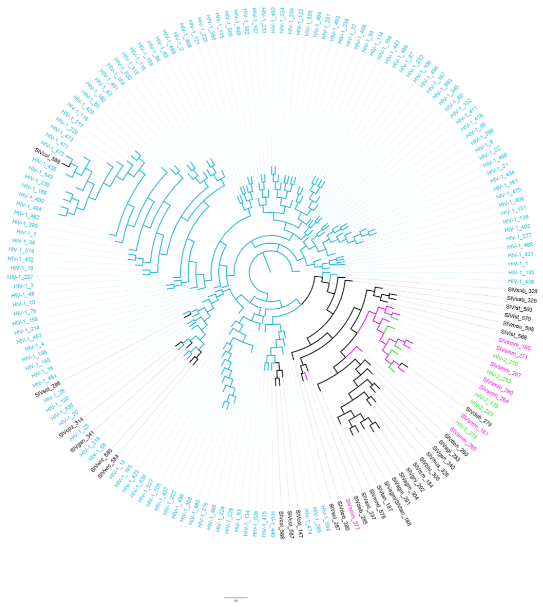

Figure S10. S2 tree comparison. Phylogenetic tree of S2 constructed using the maximum likelihood (ML) method. The tree shows a comparable grouping for HIV-1, HIV-2 and SIVs. Viruses color code: HIV-1 azure, HIV-2 green, SIVsmm purple (and other SIVs in black).

Table S1. Clusters dataset of 37-52 p17 fragments. The table shows the dataset used for phylogenetic analysis. For each cluster is shown: cluster number, aa fragment sequence, virus strain, number of sequences included in each cluster and percentage of sequence per cluster. In the table 37-52 fragments sequences belonging to each cluster are associated with a unique virus (HIV-1, HIV-2 or SIV) with accuracy close to 100%.

Cluster number	37-52 p17 fragment sequence	Virus strain	Number of sequences for each cluster	%
1	ASRELERFAVNPGLLE	HIV-1	13732	41,386
2	ASRELERFALNPGLLE	HIV-1	10949	32,999
3	ASRELERFALNPSLLE	HIV-1	2125	6,404
4	ASRELERFAINPGLLE	HIV-1	988	2,978
5	ASRELDRFALNPGLLE	HIV-1	456	1,374
6	ASRELEKFALNPGLLE	HIV-1	399	1,203
7	ASRELERFALNPDLLE	HIV-1	371	1,118
8	ASRELERFALDPGLLE	HIV-1	306	0,922
9	ASRELDRFALNPSLLE	HIV-1	297	0,895
10	ASRELERFAVNPSLLE	HIV-1	216	0,651
11	ASRELERFAVNPSLME	HIV-1	197	0,594
12	AANELDRFGLAESLLE	HIV-2/SIVsmm	180	0,542
13	ASRELEKFALNPDLLE	HIV-1	116	0,35
14	ASRELDRFAVNPGLLE	HIV-1	92	0,277
15	ASRELERFAVNSGLLE	HIV-1	92	0,277
16	ASRELERFALNSGLLE	HIV-1	83	0,25
17	ASRELDRFAINPGLLE	HIV-1	77	0,232
18	ASRELERFAVNPGLLD	HIV-1	60	0,181
19	ASRELERFALNPNLLE	HIV-1	57	0,172

20	ASRELERFALNSSLLE	HIV-1	56	0,169
21	ASRELERFALDPSLLE	HIV-1	54	0,163
22	ASRELERFAVDPGLLE	HIV-1	52	0,157
23	ASRELERFALSSGLLE	HIV-1	48	0,145
24	ASREMERFALNPSLLE	HIV-1	48	0,145
25	ASRELERYALNPGLLE	HIV-1	46	0,139
26	AGRELERFALNPGLLE	HIV-1	45	0,136
27	ASKELERFALNPGLLE	HIV-1	41	0,124
28	ASRELERFALNSDLLE	HIV-1	41	0,124
29	ASRELGRFALNPGLLE	HIV-1	41	0,124
30	ASRELEKFALNPSLLE	HIV-1	39	0,118
31	ASRELERFALNPGLLD	HIV-1	37	0,112
32	AGKEMERFGLHEKLLE	SIVver/SIVgrv/SIVtan/SIVmal	36	0,108
33	ASKELERFAVNPGLLE	HIV-1	36	0,108
34	ASRELERFALNPELLE	HIV-1	36	0,108
35	ASREMERFALNPGLLE	HIV-1	34	0,102
36	VSRELERFAVNPGLLE	HIV-1	34	0,102
37	ASRELERFSLNPGLLE	HIV-1	32	0,096
38	AANELDRFGLAESLLD	SIVsmm	30	0,09
39	ASRELERFSINPGLLE	HIV-1	29	0,087
40	AGRELERFAVNPGLLE	HIV-1	25	0,075
41	ASRELERFAVNPGLME	HIV-1	25	0,075
42	AGRELERFALNPSLLE	HIV-1	23	0,069
43	ASRELDRFALNPDLLE	HIV-1	23	0,069
44	ASRELEKFAINPGLLE	HIV-1	20	0,06
45	ASRELERFALNPGFLE	HIV-1	20	0,06
46	ASRELERFAVNPGLIE	HIV-1	20	0,06

47	ASRELERYAVNPGLLE	HIV-1	20	0,06
48	ASRELERFAINPSLLE	HIV-1	18	0,054
49	ASRELERFALNPGLME	HIV-1	18	0,054
50	ASRELDRFALNSGLLE	HIV-1	17	0,051
51	ASRELGRFALNSGLLE	HIV-1	17	0,051
52	ASREVERFALNPGLLE	HIV-1	17	0,051
53	ASNELERFALNPGLLE	HIV-1	15	0,045
54	ASRELEKFSLNPGLLE	HIV-1	15	0,045
55	ASRELENFALNPGLLE	HIV-1	15	0,045
56	ASRELERFAIDPGLLE	HIV-1	15	0,045
57	ASKELEKFALNPGLLE	HIV-1	14	0,042
58	ASQELERFALNPGLLE	HIV-1	14	0,042
59	ASRELDRFALNPGLLD	HIV-1	14	0,042
60	ASRELERFACNPGLLE	HIV-1	14	0,042
61	ASRELERFALNPGPLE	HIV-1	14	0,042
62	ASRELERFAVNPDLLE	HIV-1	14	0,042
63	ASRELERFAVNAGLLE	HIV-1	13	0,039
64	ASRQLERFALNPSLLE	HIV-1	13	0,039
65	ASRELEKFSINPGLLE	HIV-1	12	0,036
66	ASRELEQFAINPGLLE	HIV-1	11	0,033
67	ASRELERFACNPGLME	HIV-1/SIVcpz	11	0,033
68	ASRELERFALDSGLLE	HIV-1	11	0,033
69	ASRELERFALNPALLE	HIV-1	11	0,033
70	ASREMEKFALNSDLLE	HIV-1	11	0,033
71	ASGELERFAVNPGLLE	HIV-1	10	0,03
72	ASKELERFAINPGLLE	HIV-1	10	0,03
73	ASRELERFACNPGLMD	HIV-1/SIVcpz	10	0,03

74	ASRELGRFAVNPGLLE	HIV-1	10	0,03
75	ASREMERFALNSDLLE	HIV-1	10	0,03
76	ASRELDRFALDPGLLE	HIV-1	9	0,027
77	ASRELEQFAVNPGLLE	HIV-1	9	0,027
78	ASRELERFAVNPALLE	HIV-1	9	0,027
79	ASRELERVALNPSLLE	HIV-1	9	0,027
80	ASREVERFALNPELLE	HIV-1	9	0,027
81	AANELDRFGLADSLLE	SIVsmm	8	0,024
82	AGSELQRFAMNPGLME	SIVcpz	8	0,024
83	ASGELERFALNPGLLE	HIV-1	8	0,024
84	ASRELEGFALNPGLLE	HIV-1	8	0,024
85	ASRELERFALDPDLLE	HIV-1	8	0,024
86	ASRELERFAVNPCLLE	HIV-1	8	0,024
87	ASRELERFSINPSLLE	HIV-1	8	0,024
88	ASRELGRFALNPSLLE	HIV-1	8	0,024
89	ASRELSRFALNPSLLE	HIV-1	8	0,024
90	ASREMEKFSLNPGLLE	HIV-1	8	0,024
91	ASRGLERFALNPGLLE	HIV-1	8	0,024
92	ATNELDRFGLAESLLE	SIVsmm	8	0,024
93	ASRELERFALNPGLLG	HIV-1	7	0,021
94	ASRELERFALNPSPLE	HIV-1	7	0,021
95	ASRELERFAVNPGLLK	HIV-1	7	0,021
96	ASRELERFAVNPGPLE	HIV-1	7	0,021
97	ASRELERLAVNPGLLE	HIV-1	7	0,021
98	ASRELERYALNPSLLE	HIV-1	7	0,021
99	ASRGLERFAVNPGLLE	HIV-1	7	0,021
100	AGKEMERFGLHERLLE	SIVgrv/SIVver	6	0,018
101	ASRELDKFALNPGLLE	HIV-1	6	0,018

102	ASRELDRFACNPELLE	HIV-1	6	0,018
103	ASRELEQFALNPGLLE	HIV-1	6	0,018
104	ASRELERFACNPELME	SIVgor	6	0,018
105	ASRELERFALNHSLLE	HIV-1	6	0,018
106	ASRELERFSVNPGLLE	HIV-1	6	0,018
107	ASRELERFTLNPGLLE	HIV-1	6	0,018
108	ASRELNRFALNPSLLE	HIV-1	6	0,018
109	AARELDRFGLAESLLE	SIVsmm	5	0,015
110	AGRELERFAINPGLLE	HIV-1	5	0,015
111	AGRELERFALNPDLLE	HIV-1	5	0,015
112	ASKELERFALNPSLLE	HIV-1	5	0,015
113	ASKELERFGLSDSLLE	SIVmus/SIV asc	5	0,015
114	ASRELEKFSINPDLLA	HIV-1	5	0,015
115	ASRELERFALHPGLLE	HIV-1	5	0,015
116	ASRELERFALNPCLLE	HIV-1	5	0,015
117	ASRELERFALNPGLLK	HIV-1	5	0,015
118	ASRELERFALSPGLLE	HIV-1	5	0,015
119	ASRELERFAVNPGLLG	HIV-1	5	0,015
120	ASRELERFAVNSSLLE	HIV-1	5	0,015
121	ASRELERFAVYPGLLE	HIV-1	5	0,015
122	ASRELERFVLNPGLLE	HIV-1	5	0,015
123	ASRELERLSINPGLLE	HIV-1	5	0,015
124	TSRELERFAVNPGLLE	HIV-1	5	0,015
125	VAKELDRFGLHERLLE	SIVdrl	5	0,015
126	VSKELDRFGLHEKLLE	SIVmnd-2	5	0,015
127	VSRELERYAVNPGLLE	HIV-1	5	0,015
128	AGRELEKFALNPGLLE	HIV-1	4	0,012
129	ASRELDRFALNSDLLE	HIV-1	4	0,012

130	ASRELEGFALNPSLLE	HIV-1	4	0,012
131	ASRELERFAANPGLLE	HIV-1	4	0,012
132	ASRELERFACNPELLE	HIV-1/SIVgor	4	0,012
133	ASRELERFAINPSFLE	HIV-1	4	0,012
134	ASRELERFALNAGLLE	HIV-1	4	0,012
135	ASRELERFALNASLLE	HIV-1	4	0,012
136	ASRELERFALNPEFLE	HIV-1	4	0,012
137	ASRELERFALNPSLLD	HIV-1	4	0,012
138	ASRELERFAPNPGLLE	HIV-1	4	0,012
139	ASRELERFAVNPGFLE	HIV-1	4	0,012
140	ASRELERFSLNSGLLE	HIV-1	4	0,012
141	ASRELERSALNPGLLE	HIV-1	4	0,012
142	ASRELERYAINPGLLE	HIV-1	4	0,012
143	ASRELERYSLNPGLLE	HIV-1	4	0,012
144	ASRELKRFALNPGLLE	HIV-1	4	0,012
145	ASREPERFALNPGLLE	HIV-1	4	0,012
146	ASRQLERFALNPGLLE	HIV-1	4	0,012
147	MCTEVERLQLNIELLK	SIVcol	4	0,012
148	AANELDRYGLAESLLE	SIVsmm	3	0,009
149	AGRELERYAVNPGLLE	HIV-1	3	0,009
150	AQQELERFAVNPGLLE	HIV-1	3	0,009
151	ARRELERFAVNPGLLE	HIV-1	3	0,009
152	ASKELENFALNPGLLE	HIV-1	3	0,009
153	ASRELDRFSLNPGLLE	HIV-1	3	0,009
154	ASRELEGFAVNPGLLE	HIV-1	3	0,009
155	ASRELEKFSINPSLLE	HIV-1	3	0,009
156	ASRELEKFSLNPDLLE	HIV-1	3	0,009
157	ASRELEQFAVNSGLLK	HIV-1	3	0,009

158	ASRELERFAFNPSLLE	HIV-1	3	0,009
159	ASRELERFAINPCLLE	HIV-1	3	0,009
160	ASRELERFAINPDLLE	HIV-1	3	0,009
161	ASRELERFALEPSLLE	HIV-1	3	0,009
162	ASRELERFALNPGVLE	HIV-1	3	0,009
163	ASRELERFALNPSLME	HIV-1	3	0,009
164	ASRELERFALNPVLLE	HIV-1	3	0,009
165	ASRELERFAVNHGLLE	HIV-1	3	0,009
166	ASRELERFAVNPGLSE	HIV-1	3	0,009
167	ASRELERFPLNPGLLE	HIV-1	3	0,009
168	ASRELERFSVTPGLLE	HIV-1	3	0,009
169	ASRELERSALNPSLLE	HIV-1	3	0,009
170	ASRELERSAVNPGLLE	HIV-1	3	0,009
171	ASRELETFALNPGLLE	HIV-1	3	0,009
172	ASRELGRFAINPGLLE	HIV-1	3	0,009
173	ASREMERFALNSGLLE	HIV-1	3	0,009
174	ASREPERFALNPSLLE	HIV-1	3	0,009
175	ASREVERFAVNPGLLE	HIV-1	3	0,009
176	ASRGLERFALNPSLLE	HIV-1	3	0,009
177	ASRKLDRFALNPALLE	HIV-1	3	0,009
178	TSRELERFALNPGLLE	HIV-1	3	0,009
179	AANELDKFGLAESLLE	HIV-2	2	0,006
180	AANELDRFGLAENLLE	SIVsmm	2	0,006
181	AANELDRFGLSEGLLE	SIVsmm	2	0,006
182	AANELDRFGLTESLLE	HIV-2	2	0,006
183	AANKLDRFGLAESLLE	HIV-2	2	0,006
184	ACKKLNKFGLSDHLLE	SIVrcm	2	0,006
185	ACRELERFAVNPGLLE	HIV-1	2	0,006
186	ACRELERFGLSDTLLE	SIVrcm	2	0,006

187	AGKEMERFGLHDKLLE	SIVtan	2	0,006
188	AGKEMERFGLHDRLLE	SIVagm/SIVtan	2	0,006
189	AGREMERFGLHEKLLE	SIVtan/SIVagm	2	0,006
190	ANRELERFALNPGLLE	HIV-1	2	0,006
191	ASKELERFACNPELME	SIVgor	2	0,006
192	ASKELGRFALNPGLLE	HIV-1	2	0,006
193	ASKEMERFGLSDALLE	SIVtal	2	0,006
194	ASMELERFALNPSLLE	HIV-1	2	0,006
195	ASRDLERFALNPSLLE	HIV-1	2	0,006
196	ASRELARFALNPGLLE	HIV-1	2	0,006
197	ASRELDRFACNPGLME	SIVcpz	2	0,006
198	ASRELDRFAINSGLLE	HIV-1	2	0,006
199	ASRELDRFALNPELLE	HIV-1	2	0,006
200	ASRELDRFALNPGFLE	HIV-1	2	0,006
201	ASRELDRFALNPGLLK	HIV-1	2	0,006
202	ASRELDRFALNPGLME	HIV-1	2	0,006
203	ASRELDRFALNPNLLE	HIV-1	2	0,006
204	ASRELDRFAVNPSLME	HIV-1	2	0,006
205	ASRELDRYALNPDLLE	HIV-1	2	0,006
206	ASRELEGFALNSSLLE	HIV-1	2	0,006
207	ASRELEHFAINPGLLE	HIV-1	2	0,006
208	ASRELEKFALNPGLLK	HIV-1	2	0,006
209	ASRELEKFAVNPGLLE	HIV-1	2	0,006
210	ASRELEKYSINPGLLE	HIV-1	2	0,006
211	ASRELENFALNPSLLE	HIV-1	2	0,006
212	ASRELENFALNSGLLE	HIV-1	2	0,006
213	ASRELERFACDPGLME	SIVcpz	2	0,006

214	ASRELERFAFNPGLLE	HIV-1	2	0,006
215	ASRELERFAINPGLLK	HIV-1	2	0,006
216	ASRELERFAINSGLLE	HIV-1	2	0,006
217	ASRELERFAISPGLLE	HIV-1	2	0,006
218	ASRELERFALDSSLLE	HIV-1	2	0,006
219	ASRELERFALNPGLSE	HIV-1	2	0,006
220	ASRELERFALNPGLVE	HIV-1	2	0,006
221	ASRELERFALNPSFLE	HIV-1	2	0,006
222	ASRELERFALNRDLLE	HIV-1	2	0,006
223	ASRELERFAVHPGLLE	HIV-1	2	0,006
224	ASRELERFAVNNGLLE	HIV-1	2	0,006
225	ASRELERFAVNPGHLE	HIV-1	2	0,006
226	ASRELERFAVNPGLVE	HIV-1	2	0,006
227	ASRELERFAVNPNLLE	HIV-1	2	0,006
228	ASRELERFAVNTGLLE	HIV-1	2	0,006
229	ASRELERFAVSPGLLE	HIV-1	2	0,006
230	ASRELERFAVTPGLLE	HIV-1	2	0,006
231	ASRELERFILNPGLLE	HIV-1	2	0,006
232	ASRELERFSLNPSLLE	HIV-1	2	0,006
233	ASRELERFTVNPGLLE	HIV-1	2	0,006
234	ASRELERFVINPSLLE	HIV-1	2	0,006
235	ASRELERFVLNPSLLE	HIV-1	2	0,006
236	ASRELERFVVNPGLLE	HIV-1	2	0,006
237	ASRELERLALNAGLLE	HIV-1	2	0,006
238	ASRELERLSLNPSLLE	HIV-1	2	0,006
239	ASRELERYSINPGLLE	HIV-1	2	0,006
240	ASRELGRFALNSDLLE	HIV-1	2	0,006
241	ASRELKRFAVNPGLLE	HIV-1	2	0,006
242	ASRELSRFALNPDLLE	HIV-1	2	0,006

243	ASRELSRFALNPGLLE	HIV-1	2	0,006
244	ASREMEKFALNPGLLE	HIV-1	2	0,006
245	ASREMERFALNPDLLE	HIV-1	2	0,006
246	ASREPERFAVNPGLLE	HIV-1	2	0,006
247	ASREQERFALNPGLLE	HIV-1	2	0,006
248	ASREVERFALDPGLLE	HIV-1	2	0,006
249	ASRGLDRFALNPSLLE	HIV-1	2	0,006
250	ASRKLERFAVNPGLLE	HIV-1	2	0,006
251	ASRKLERFAVNPGLLK	HIV-1	2	0,006
252	AVNELDRFGLAESLLE	HIV-2	2	0,006
253	AVNELDRFGLAETLLE	HIV-2	2	0,006
254	PSRELEQFAVNSGLLK	HIV-1	2	0,006
255	PSRELERFALNPGLLE	HIV-1	2	0,006
256	SSRELERFAVNPGLLE	HIV-1	2	0,006
257	TSRELERFAINPGLLE	HIV-1	2	0,006
258	VSRELERFAVNHGLLE	HIV-1	2	0,006
259	AAKELDRFGLAESLLE	SIVsmm	1	0,003
260	AAKELDRFGLSDSLLE	SIVden	1	0,003
261	AANELDGFGLAESLLR	SIVsmm	1	0,003
262	AANELDKFGLAESLLD	SIVsmm	1	0,003
263	AANELDKFGLTESLLE	HIV-2	1	0,003
264	AANELDRFGFAESLLE	SIVsmm	1	0,003
265	AANELDRFGLADNLLE	SIVsmm	1	0,003
266	AANELDRFGLADSLLG	SIVsmm	1	0,003
267	AANELDRFGLAEGLLE	SIVsmm	1	0,003
268	AANELDRFGLAESLVE	SIVsmm	1	0,003
269	AANELDRFGLAETLLE	SIVsmm	1	0,003
270	AANELDRFRLAENLLE	HIV-2	1	0,003
271	AANELDRFRLAERLLE	SIVsmm	1	0,003

272	AANELERFGLAENLLR	SIVsmm	1	0,003
273	AANELGKFGLAESLLE	HIV-2	1	0,003
274	AANELNRFGLSESPVE	HIV-2	1	0,003
275	AANGLDRFGLAESLLD	SIVsmm	1	0,003
276	AANGLDRFGLAESLLE	SIVsmm	1	0,003
277	AANGLDRFGLPGKLLG	SIVsmm	1	0,003
278	AANRIGRFGLAESLLE	SIVsmm	1	0,003
279	AARELDRFGLSEALLE	SIVdeb	1	0,003
280	AARELDRFGSAESLLE	SIVsmm	1	0,003
281	AASELDRFGLAESLLA	SIVsmm	1	0,003
282	ACRELEQFAVNPGLLE	HIV-1	1	0,003
283	ACRELERFGLSDTLLD	SIVagi	1	0,003
284	AENELDRFGLAESLLE	SIVsmm	1	0,003
285	AGKELDRFGLAAALLE	SIVdeb	1	0,003
286	AGKELDRFGLNKELLR	SIVsol	1	0,003
287	AGKELDRFGLSADLLR	SIVsol	1	0,003
288	AGKELDRFGLSANLLE	SIVtan	1	0,003
289	AGKELDRFGLSDQLLE	SIVsyk	1	0,003
290	AGKELERFALNPGLLE	HIV-1	1	0,003
291	AGKEMERFGLHQKLLE	SIVagm	1	0,003
292	AGKKMDRFGLHEKLLE	SIVgrv	1	0,003
293	AGKKMERFGLHEKLLE	SIVtan	1	0,003
294	AGNELQRFALNPGLME	SIVcpz	1	0,003
295	AGNQLERFALNPGLME	SIVcpz	1	0,003
296	AGRELDRFALDPGLLE	HIV-1	1	0,003
297	AGRELDRFALNPSLLE	HIV-1	1	0,003
298	AGRELDRFAMDPGLLE	HIV-1	1	0,003
299	AGRELERFALDPGLLE	HIV-1	1	0,003
300	AGRELERFALDSGLLE	HIV-1	1	0,003

301	AGRELERFAVNAGLLE	HIV-1	1	0,003
302	AGRELERFAVNSGLLE	HIV-1	1	0,003
303	AGRELGRFAINPGLLE	HIV-1	1	0,003
304	AGREMERFGLHERLLE	SIVagm	1	0,003
305	AGSELERFAMNPGLME	SIVcpz	1	0,003
306	AKKELDRFGLSDQLLE	SIVblu	1	0,003
307	AKKELDRFGLSDQLME	SIVsyk	1	0,003
308	ANRELDKFAFNRGVFG	HIV-1	1	0,003
309	ANRELEKFALNPDLLD	HIV-1	1	0,003
310	ANRELERFAINPGLLE	HIV-1	1	0,003
311	ANRELERFAVNPGLLE	HIV-1	1	0,003
312	APNELDRFGLAESLLE	SIVsmm	1	0,003
313	ARRELEKSALNPSLLE	HIV-1	1	0,003
314	ARSELQRFALSSSLLE	SIVcpz	1	0,003
315	ASGEGERFALNPTLLK	HIV-1	1	0,003
316	ASGELEKFSLNPGLLE	HIV-1	1	0,003
317	ASGELERFAINPGLLE	HIV-1	1	0,003
318	ASGELERFAINPSFLE	HIV-1	1	0,003
319	ASGELERFALNPSLLE	HIV-1	1	0,003
320	ASGELERFALNSGLLE	HIV-1	1	0,003
321	ASGELERFAPNPGLLE	HIV-1	1	0,003
322	ASGELGRFALNPSLLE	HIV-1	1	0,003
323	ASKELDRFALNPGLLE	HIV-1	1	0,003
324	ASKELDRFALNPSLLE	HIV-1	1	0,003
325	ASKELDRFGLSANLLE	SIVsab	1	0,003
326	ASKELDRFGLSDALLE	SIVmus	1	0,003
327	ASKELDRFGLSDSLLE	SIVmus	1	0,003
328	ASKELDRFSLSANLLE	SIVsab	1	0,003
329	ASKELDRYAVNPGLLE	HIV-1	1	0,003

330	ASKELEHFALNPGLLE	HIV-1	1	0,003
331	ASKELERFAIDPGLLE	HIV-1	1	0,003
332	ASKELERFAINPGPLE	HIV-1	1	0,003
333	ASKELERFALDPSLLE	HIV-1	1	0,003
334	ASKELERFALNPDLLE	HIV-1	1	0,003
335	ASKELERFAVNPGLLG	HIV-1	1	0,003
336	ASKELERFAVNSRLLE	HIV-1	1	0,003
337	ASKELERFGLADSLLE	SIVasc	1	0,003
338	ASKELERLALNPGLLE	HIV-1	1	0,003
339	ASKELERLAVNPGLLE	HIV-1	1	0,003
340	ASKELERYALSDALLE	SIVgsn	1	0,003
341	ASKELERYALSGSLLE	SIVgsn	1	0,003
342	ASKEVERFTLNPGLFE	HIV-1	1	0,003
343	ASMELERFALNPGLLE	HIV-1	1	0,003
344	ASMELERFAVNPGLLE	HIV-1	1	0,003
345	ASQELEKFKVNPGLLG	HIV-1	1	0,003
346	ASQELERFALNPSLLE	HIV-1	1	0,003
347	ASQELERFAVNPGLLE	HIV-1	1	0,003
348	ASQELERLALNPGLLE	HIV-1	1	0,003
349	ASQELSRFALNPSLLE	HIV-1	1	0,003
350	ASRALERFAVNPGLLE	HIV-1	1	0,003
351	ASRAVEQLAVNPGLLE	HIV-1	1	0,003
352	ASREAERFALNPGLLE	HIV-1	1	0,003
353	ASREAERYALNPGLLE	HIV-1	1	0,003
354	ASRECERFAVNPGLLE	HIV-1	1	0,003
355	ASREEERFAINPGLLE	HIV-1	1	0,003
356	ASREFERFALNPGLLE	HIV-1	1	0,003
357	ASREFERFAVNPGLLE	HIV-1	1	0,003
358	ASREGDRLAVNPSLLE	HIV-1	1	0,003

359	ASREIERFALNPGLLE	HIV-1	1	0,003
360	ASREIERFAVNPGLLE	HIV-1	1	0,003
361	ASREIERFAVYPGLFE	HIV-1	1	0,003
362	ASREIERFSINPGLLE	HIV-1	1	0,003
363	ASRELARFALNPDLLE	HIV-1	1	0,003
364	ASRELDKFAINPGLLE	HIV-1	1	0,003
365	ASRELDKFSINPGLLE	HIV-1	1	0,003
366	ASRELDKFSLHPGFLE	HIV-1	1	0,003
367	ASRELDRFACNPGLMD	HIV-1	1	0,003
368	ASRELDRFAINPGLIE	HIV-1	1	0,003
369	ASRELDRFALDSSLLE	HIV-1	1	0,003
370	ASRELDRFALNAGLLE	HIV-1	1	0,003
371	ASRELDRFALNPDLLG	HIV-1	1	0,003
372	ASRELDRFALNPGLIE	HIV-1	1	0,003
373	ASRELDRFALNPSLLD	HIV-1	1	0,003
374	ASRELDRFALNPSLLG	HIV-1	1	0,003
375	ASRELDRFALNRGILE	HIV-1	1	0,003
376	ASRELDRFALNSSLLE	HIV-1	1	0,003
377	ASRELDRFATNPGLLE	HIV-1	1	0,003
378	ASRELDRFAVNPGLLG	HIV-1	1	0,003
379	ASRELDRFAYNPELLE	HIV-1	1	0,003
380	ASRELDRFGLAGALLE	SIVdeb	1	0,003
381	ASRELDRFGLSDSLLE	SIVmus	1	0,003
382	ASRELDRFTLNPSLLE	HIV-1	1	0,003
383	ASRELDRYACNPELLE	HIV-1	1	0,003
384	ASRELDRYALNPGLLD	HIV-1	1	0,003
385	ASRELEAFALNLGLLA	HIV-1	1	0,003
386	ASRELEGFALDPSLLE	HIV-1	1	0,003
387	ASRELEGFAVNPSLLE	HIV-1	1	0,003

388	ASRELEHFSINPGLLE	HIV-1	1	0,003
389	ASRELEIFALNPSLLE	HIV-1	1	0,003
390	ASRELEKFAFNPSLLE	HIV-1	1	0,003
391	ASRELEKFAINPDLLE	HIV-1	1	0,003
392	ASRELEKFAINPGLIE	HIV-1	1	0,003
393	ASRELEKFALDPSLLE	HIV-1	1	0,003
394	ASRELEKFALNPCLLE	HIV-1	1	0,003
395	ASRELEKFALNPDPLE	HIV-1	1	0,003
396	ASRELEKFALNPGLME	HIV-1	1	0,003
397	ASRELEKFALNPGLSE	HIV-1	1	0,003
398	ASRELEKFALNSDLLE	HIV-1	1	0,003
399	ASRELEKFALSPDLLE	HIV-1	1	0,003
400	ASRELEKFALTPGLLE	HIV-1	1	0,003
401	ASRELEKFSINPDLLE	HIV-1	1	0,003
402	ASRELEKFSPNPGLLE	HIV-1	1	0,003
403	ASRELEKFTLNPGLLE	HIV-1	1	0,003
404	ASRELEKFVLNPGLLE	HIV-1	1	0,003
405	ASRELEKSALNPGVLE	HIV-1	1	0,003
406	ASRELEKSALNPSLLE	HIV-1	1	0,003
407	ASRELEKYSLNPDLLE	HIV-1	1	0,003
408	ASRELENFACDPGLLE	HIV-1	1	0,003
409	ASRELENFALNPDLLE	HIV-1	1	0,003
410	ASRELENFSLNPGLLE	HIV-1	1	0,003
411	ASRELENYACDPELLD	HIV-1	1	0,003
412	ASRELEQFAINPGLIE	HIV-1	1	0,003
413	ASRELEQFALNPSLLE	HIV-1	1	0,003
414	ASRELEQFSINPGLLE	HIV-1	1	0,003
415	ASRELEQYAINPGLLE	HIV-1	1	0,003
416	ASRELERCAINPGLLE	HIV-1	1	0,003

417	ASRELERFACDPELLE	HIV-1	1	0,003
418	ASRELERFACNPSLLE	HIV-1	1	0,003
419	ASRELERFACNPSLME	SIVcpz	1	0,003
420	ASRELERFAFNPGLFE	HIV-1	1	0,003
421	ASRELERFAGNPGLLE	HIV-1	1	0,003
422	ASRELERFAIDPGLLD	HIV-1	1	0,003
423	ASRELERFAINHGLLE	HIV-1	1	0,003
424	ASRELERFAINPGLIE	HIV-1	1	0,003
425	ASRELERFAINPGLLD	HIV-1	1	0,003
426	ASRELERFAINPGLLG	HIV-1	1	0,003
427	ASRELERFAINPGLLQ	HIV-1	1	0,003
428	ASRELERFAINPGLME	HIV-1	1	0,003
429	ASRELERFALAPGLLE	HIV-1	1	0,003
430	ASRELERFALDPGFLE	HIV-1	1	0,003
431	ASRELERFALDPGLLD	HIV-1	1	0,003
432	ASRELERFALDPGPLE	HIV-1	1	0,003
433	ASRELERFALDPSPLE	HIV-1	1	0,003
434	ASRELERFALKPSLLE	HIV-1	1	0,003
435	ASRELERFALLSGLLE	HIV-1	1	0,003
436	ASRELERFALNANLLE	HIV-1	1	0,003
437	ASRELERFALNHDLLE	HIV-1	1	0,003
438	ASRELERFALNHGLLE	HIV-1	1	0,003
439	ASRELERFALNLGLLE	HIV-1	1	0,003
440	ASRELERFALNLSLLE	HIV-1	1	0,003
441	ASRELERFALNPDILE	HIV-1	1	0,003
442	ASRELERFALNPDLIE	HIV-1	1	0,003
443	ASRELERFALNPDLLD	HIV-1	1	0,003
444	ASRELERFALNPDLLG	HIV-1	1	0,003
445	ASRELERFALNPDLME	HIV-1	1	0,003

446	ASRELERFALNPDLVE	HIV-1	1	0,003
447	ASRELERFALNPGCLE	HIV-1	1	0,003
448	ASRELERFALNPGFLD	HIV-1	1	0,003
449	ASRELERFALNPGILE	HIV-1	1	0,003
450	ASRELERFALNPGRLE	HIV-1	1	0,003
451	ASRELERFALNPILLE	HIV-1	1	0,003
452	ASRELERFALNPRVFV	HIV-1	1	0,003
453	ASRELERFALNPSLFE	HIV-1	1	0,003
454	ASRELERFALNPSLIE	HIV-1	1	0,003
455	ASRELERFALNPSLLK	HIV-1	1	0,003
456	ASRELERFALNPTLLE	HIV-1	1	0,003
457	ASRELERFALNPVFLE	HIV-1	1	0,003
458	ASRELERFALNRGLLE	HIV-1	1	0,003
459	ASRELERFALNRSLLE	HIV-1	1	0,003
460	ASRELERFALNSDLIE	HIV-1	1	0,003
461	ASRELERFALNSELLE	HIV-1	1	0,003
462	ASRELERFALSPSLLE	HIV-1	1	0,003
463	ASRELERFALTPGLLE	HIV-1	1	0,003
464	ASRELERFALTPSLLE	HIV-1	1	0,003
465	ASRELERFALYHGLLE	HIV-1	1	0,003
466	ASRELERFALYPGLLE	HIV-1	1	0,003
467	ASRELERFAMNPGLLE	HIV-1	1	0,003
468	ASRELERFASNPGLLE	HIV-1	1	0,003
469	ASRELERFAVDPALLE	HIV-1	1	0,003
470	ASRELERFAVDPSLLE	HIV-1	1	0,003
471	ASRELERFAVIPGLLE	HIV-1	1	0,003
472	ASRELERFAVISGLLE	HIV-1	1	0,003
473	ASRELERFAVKPGLLE	HIV-1	1	0,003
474	ASRELERFAVNLGLLE	HIV-1	1	0,003

475	ASRELERFAVNLVLLE	HIV-1	1	0,003
476	ASRELERFAVNPALME	HIV-1	1	0,003
477	ASRELERFAVNPCLLK	HIV-1	1	0,003
478	ASRELERFAVNPGLLA	HIV-1	1	0,003
479	ASRELERFAVNPGLLQ	HIV-1	1	0,003
480	ASRELERFAVNPGQLE	HIV-1	1	0,003
481	ASRELERFAVNPGRLE	HIV-1	1	0,003
482	ASRELERFAVNPSLIE	HIV-1	1	0,003
483	ASRELERFAVNPSLLD	HIV-1	1	0,003
484	ASRELERFAVNPSLMK	HIV-1	1	0,003
485	ASRELERFAVNRGLLE	HIV-1	1	0,003
486	ASRELERFEINPDLLE	HIV-1	1	0,003
487	ASRELERFEINPNLLE	HIV-1	1	0,003
488	ASRELERFELNPGLLE	HIV-1	1	0,003
489	ASRELERFGLNPGLLE	HIV-1	1	0,003
490	ASRELERFGLSDTLLD	SIVrcm	1	0,003
491	ASRELERFGLSDTLLE	SIVmus	1	0,003
492	ASRELERFIINPSLLE	HIV-1	1	0,003
493	ASRELERFILNPDLLE	HIV-1	1	0,003
494	ASRELERFMLNPGLLE	HIV-1	1	0,003
495	ASRELERFPLNPGLLD	HIV-1	1	0,003
496	ASRELERFPVNPGLLE	HIV-1	1	0,003
497	ASRELERFPVNPGLLK	HIV-1	1	0,003
498	ASRELERFSINPDLLE	HIV-1	1	0,003
499	ASRELERFTINPGLLE	HIV-1	1	0,003
500	ASRELERFTVDPGLLE	HIV-1	1	0,003
501	ASRELERIALNPGLLE	HIV-1	1	0,003
502	ASRELERLALNPGLLE	HIV-1	1	0,003
503	ASRELERLAVNSGLLE	HIV-1	1	0,003

504	ASRELERLSINPSLLE	HIV-1	1	0,003
505	ASRELERYACNPGLLE	HIV-1	1	0,003
506	ASRELERYAIDPGLLE	HIV-1	1	0,003
507	ASRELERYALNSDLLE	HIV-1	1	0,003
508	ASRELESFALNPGLLE	HIV-1	1	0,003
509	ASRELESFALNPSLLE	HIV-1	1	0,003
510	ASRELETFSLNPGLLE	HIV-1	1	0,003
511	ASRELGKFALNPDLLE	HIV-1	1	0,003
512	ASRELGRFALDPGLLE	HIV-1	1	0,003
513	ASRELGRFALNASLLE	HIV-1	1	0,003
514	ASRELGRFALNPDLLE	HIV-1	1	0,003
515	ASRELGRFALNPNLLE	HIV-1	1	0,003
516	ASRELGRFALNRDLLE	HIV-1	1	0,003
517	ASRELGRFAVDPGLLE	HIV-1	1	0,003
518	ASRELGRFSLNSGLLE	HIV-1	1	0,003
519	ASRELKEFALNPGLLE	HIV-1	1	0,003
520	ASRELKRFAINPVLLE	HIV-1	1	0,003
521	ASRELKRFALNPSLLE	HIV-1	1	0,003
522	ASRELNRFALDPSLLE	HIV-1	1	0,003
523	ASRELNRFALNPDLLE	HIV-1	1	0,003
524	ASRELNRFALNPGLLE	HIV-1	1	0,003
525	ASRELNRFALNPSLLG	HIV-1	1	0,003
526	ASRELQRFALNPGLLE	HIV-1	1	0,003
527	ASRELVKSALNHAVLE	HIV-1	1	0,003
528	ASRELVRFALNLVASK	HIV-1	1	0,003
529	ASRELVRFALNPSLLE	HIV-1	1	0,003
530	ASRELVRFAVNPGLLE	HIV-1	1	0,003
531	ASRELVRFSINPDLLE	HIV-1	1	0,003
532	ASRELYRFALNLGVSA	HIV-1	1	0,003

533	ASREMDRFALNPDLLE	HIV-1	1	0,003
534	ASREMDRFALNPGLLE	HIV-1	1	0,003
535	ASREMDRFALNPSLLE	HIV-1	1	0,003
536	ASREMEKFALNSGLLE	HIV-1	1	0,003
537	ASREMERFALDPGLLE	HIV-1	1	0,003
538	ASREMERFALNPGLME	HIV-1	1	0,003
539	ASREMERFALNPSLLV	HIV-1	1	0,003
540	ASREMERFAVNPGLLE	HIV-1	1	0,003
541	ASREMERSALNPSLLE	HIV-1	1	0,003
542	ASREMERYALNPGLLE	HIV-1	1	0,003
543	ASREPDRFALIPGVLE	HIV-1	1	0,003
544	ASREPDRFALNPSLLE	HIV-1	1	0,003
545	ASREPERFALDPGLLE	HIV-1	1	0,003
546	ASREPERFAVNPDLLE	HIV-1	1	0,003
547	ASREQERFALNPNLLE	HIV-1	1	0,003
548	ASRERERFAVNPALVE	HIV-1	1	0,003
549	ASRERGRFALNPGLLE	HIV-1	1	0,003
550	ASRESERFALNPGLLE	HIV-1	1	0,003
551	ASRETERFALNPGLLE	HIV-1	1	0,003
552	ASREVDRFALNPGLLE	HIV-1	1	0,003
553	ASREVEKFALNPGLLE	HIV-1	1	0,003
554	ASREVERFAINPDLLE	HIV-1	1	0,003
555	ASREVERFLINPGLLE	HIV-1	1	0,003
556	ASREWERFAVNPGVLE	HIV-1	1	0,003
557	ASRGLERFAVNPGLLG	HIV-1	1	0,003
558	ASRKLEKFALNPGLLK	HIV-1	1	0,003
559	ASRKLERFALNPDLLE	HIV-1	1	0,003
560	ASRKLERFALNPSLLE	HIV-1	1	0,003
561	ASRKLERFALNSGLLE	HIV-1	1	0,003

562	ASRQLKRFALNSGLLE	HIV-1	1	0,003
563	ASRRLERFAVNPGLLE	HIV-1	1	0,003
564	ASRVLERFALNPSLLE	HIV-1	1	0,003
565	ASSELERFAVNHGLLE	HIV-1	1	0,003
566	ASSELERFAVNPGLLE	HIV-1	1	0,003
567	ATKELDRFGLAESLLE	SIVsmm	1	0,003
568	ATKELDRFGLGAHLLE	SIVlst	1	0,003
569	ATKELDRFGLGANLLE	SIVlst	1	0,003
570	ATKELDRFGLGSQLLE	SIVlst	1	0,003
571	ATRELDRFGLGAHLLE	SIVlst	1	0,003
572	ATRELEKFALNPGLLK	HIV-1	1	0,003
573	ATRELERFALNPGLLE	HIV-1	1	0,003
574	ATRNLENFALTPGLLK	HIV-1	1	0,003
575	AVNELDRYGLAESLLE	HIV-2	1	0,003
576	AVNELDRYGLAETLLE	HIV-2	1	0,003
577	AVNELERFGLAESRLG	HIV-2	1	0,003
578	CKGELDRFGLSDKLLE	SIVmnd	1	0,003
579	EANELDRFGLAESLLE	SIVsmm	1	0,003
580	EAWWTERFAVNPGLLE	HIV-1	1	0,003
581	ESRELERFAVNPGLLE	HIV-1	1	0,003
582	FSRELDRFALNPSLLE	HIV-1	1	0,003
583	LTNELDRFGLAESLLE	SIVsmm	1	0,003
584	LVKELEKLCLDSALIS	SIVwrc	1	0,003
585	LVKELEKLCLDSTLIA	SIVwrc	1	0,003
586	LVREVERFAVNPGLLE	HIV-1	1	0,003
587	MCHEVTRLCLNVELLK	SIVcol	1	0,003
588	MCTEVNRLCLNIELLK	SIVcol	1	0,003
589	MCTEVSRCVLIFELLK	SIVcol	1	0,003
590	MCTEVTRLCLNVELLK	SIVcol	1	0,003

591	PSRELDRFAVNSGLLE	HIV-1	1	0,003
592	PSRELEKFALNPSLLE	HIV-1	1	0,003
593	PSRELEKFPINPGLLE	HIV-1	1	0,003
594	PSRELEPFALTLGLLA	HIV-1	1	0,003
595	PSRELERFALNPSLLE	HIV-1	1	0,003
596	PSRELERFAVNPGLLE	HIV-1	1	0,003
597	PSRELERFAVNSGLLE	HIV-1	1	0,003
598	SKKEMERFGLGEQLLE	SIVmon	1	0,003
599	TSRELERFALDPSLLE	HIV-1	1	0,003
600	TSRELKRFALNPSLLE	HIV-1	1	0,003
601	VSKELERYAINPGLLE	HIV-1	1	0,003
602	VSMELGRFSINPGLLE	HIV-1	1	0,003
603	VSRELDRFALNPGLLE	HIV-1	1	0,003
604	VSRELERFAINPGLLE	HIV-1	1	0,003
605	VSRELERFALNPGLLE	HIV-1	1	0,003
606	VSRELERFAVNPSLLE	HIV-1	1	0,003
607	VSRELERFPVNPGLLE	HIV-1	1	0,003
608	VSRVLERFAVNPGLLE	HIV-1	1	0,003

Table S2. Descriptive statistical analysis of S1, S2 and S3 sub-fragments. In the table are shown the results of the descriptive statistical analysis of 37-44 (S1), 45-49 (S2) and 50-52 (S3) of HIV-1 and HIV-2 p17 sub-fragments. The distribution of the three sub-fragments over the 33180 sequences is: S1-HIV-1 sub-fragment is in the majority of the HIV-1 and SIVgor, 55% of SIVcpz, and two OWM SIV sequences; S1-HIV-2 sub-fragment is in the majority of SIVsmm and HIV-2 sequences; S2-HIV-1 is in 80% of HIV-1 and in two SIVcpz sequences; S2-HIV-2 is in the majority of SIVsmm and HIV-2 sequences; S3-HIV-1 and S3-HIV-2 is mostly ubiquitous over the sequences. S2-HIV1 sub-fragment has the A[V/L]NPG pattern sequence (14187 AVNPG and 12335 ALNPG), in which the V/L amino acid variation preserves biochemical properties. Sub-fragments color code: S1 in orange, S2 in red and S3 in grey from the active fragment; S1 in azure, S2 in blue and S3 in grey from the inactive fragment.

	S1	S2	S3
Active p17 fragment	37-44ASRELERF	45-49AVNPG	50-52LLE
Inactive p17 fragment	37-44AANELDRF	45-49GLAES	50-52LLE

	Total sequences	37-44 ASRELERF	37-44 AANELDRF	45-49 A[V/L]NPG	45-49 GLAES	50-52 LLE
HIV1	32753	30079	//	26550	//	32165
SIVcpz	31	17	//	2	//	1
SIVgor	9	7	//	//	//	1
HIV2	39	//	25	//	30	37
SIVsmm	239	//	207	//	218	200
OWM SIV	109	2	//	//	//	95
